# Modulation of Gold Nanoparticle Ligand Structure–Dynamic Relationships Probed Using Solution NMR

**DOI:** 10.1021/acsnanoscienceau.3c00042

**Published:** 2023-11-08

**Authors:** Rui Huang, Stefano Fedeli, Cristina-Maria Hirschbiegel, Xianzhi Zhang, Vincent M. Rotello

**Affiliations:** Department of Chemistry, University of Massachusetts Amherst, 710 North Pleasant Street, Amherst, Massachusetts 01003, United States

**Keywords:** gold nanoparticles, nuclear magnetic resonance (NMR), T2 relaxation, supramolecular interactions, ligand dynamics

## Abstract

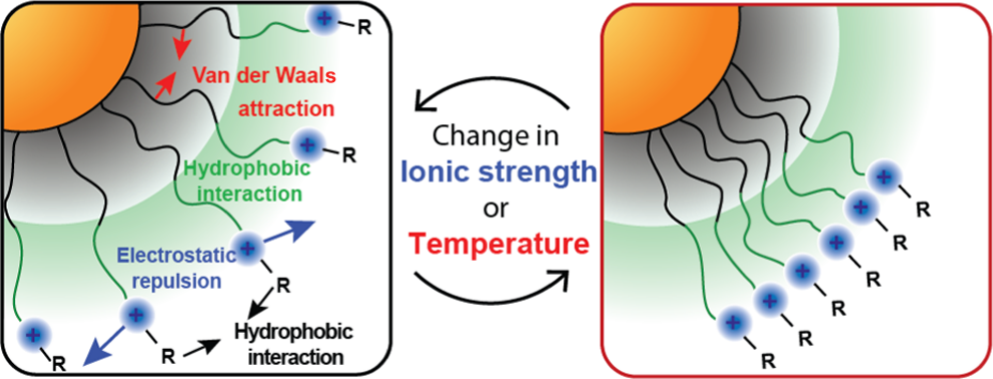

Ligand dynamics plays a critical role in the chemical and biological properties of gold nanoparticles (AuNPs). In this study, ligands featuring hydrophobic alkanethiol interiors and hydrophilic shells were used to systematically examine the effects of ligand headgroups on the ligand dynamics. Solution nuclear magnetic resonance (NMR) spectroscopy provided quantitative insight into the monolayer ligand dynamics. Notably, the introduction of hydrophobic moieties to the cationic headgroups significantly decreased ligand conformational mobility; however, variations in hydrophobicity among these moieties had a limited effect on this reduction. Further examination of ligand dynamics under various physiological conditions, including ionic strength and temperature, showed that ligands bound to the AuNP surface become less conformationally mobile with an increase in ionic strength or decreasing temperature. This exploration of ligand dynamics provides insight into designing nanoparticles tailored to specific biological applications.

## 1 Introduction

Nanomaterials are versatile tools for a diverse array of biomedical applications, ranging from biosensing^1−5^ to bioimaging^6−9^ and therapeutics.^10−16^ Gold nanoparticles (AuNPs) feature ease of functionalization,^17−20^ high biocompatibility,^21−23^ and an intrinsically nontoxic core,^24−26^ making them important biomedical materials. AuNPs are usually functionalized with an organic ligand shell to prevent agglomeration, oxidation, and degradation and to improve colloidal stability.^27−29^ AuNPs are commonly decorated using thiols that can be easily conjugated to the gold core through Au–S bonds, yielding a stable ligand shell around the AuNP.^30,31^ The electronic and optical characteristics, stability, and biocompatibility of AuNPs are determined by the molecular composition and conformation of the ligand shells, including the packing density and intramolecular dynamics of surface-bound thiolated ligands.^32,33^

Solution nuclear magnetic resonance spectroscopy (NMR) is an effective tool to elucidate the surface chemistry of ligand shells on AuNPs.^34−36^ The structure and dynamics of ligands attached to AuNPs can be analyzed qualitatively and quantitatively in a noninvasive manner, making it a versatile technique for characterizing the ligand structure–dynamic relationships.^37,38^

**Figure 1:**
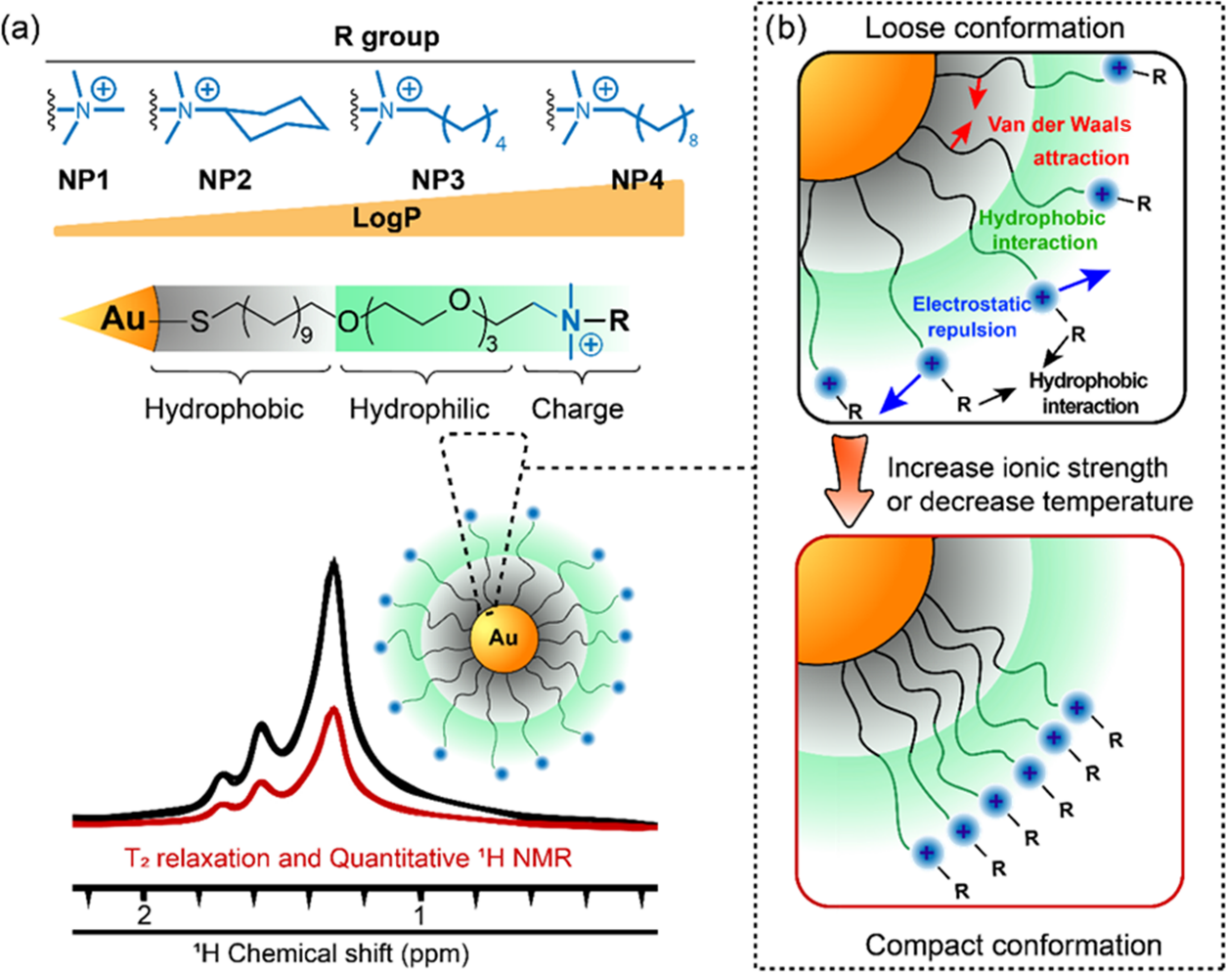
(a) Chemical structure of AuNPs including a hydrophobic alkane chain, an ethylene glycol spacer, and a headgroup. LogP represents the calculated relative hydrophobicity of the functional headgroups (NP1 ≈ 0.02, NP2 ≈ 2.1, NP3 ≈ 2.7, NP4 ≈ 4.1).^39^ Log *P* values were estimated by using ChemDraw Prime. (b) Schematic representation of ligand dynamics under varying ionic strengths and temperatures.

In this study, we fabricated a family of AuNPs to systematically investigate the effects of the ligand headgroup structure on the conformation of surface ligands on AuNPs (Figure 1a). These AuNPs featured a hydrophobic alkanethiol interior and a hydrophilic exterior shell composed of a tetra (ethylene glycol) (TEG) spacer. This common scaffold facilitates the systematic determination of the role of the headgroup on ligand dynamics by focusing on changes occurring with each particle. Quantitative NMR and T2 relaxation experiments were utilized to quantify the conformational mobility of the surface ligands. The results revealed that modifying the cationic tetramethylammonium headgroups with hydrophobic moieties considerably increased chain packing despite the intervening TEG linker. This headgroup-induced chain packing became more prominent with a decreased temperature or increased ionic strength (Figure 1b).

## 2 Results and Discussion

### 2.1 Fabrication and Characterization of the AuNPs

**Figure 2:**
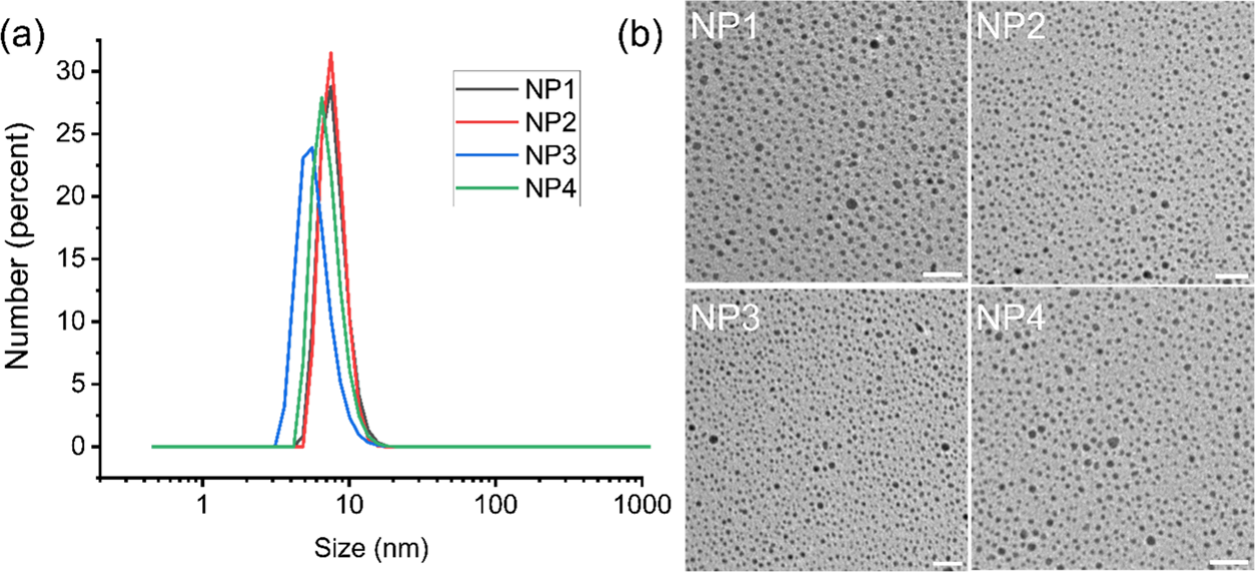
(a) Dynamic light scattering (DLS) measurements of four AuNPs dispersed in D_2_O at room temperature. (b) Transmission electron microscopy (TEM) images of four AuNP nanoparticles. The scale bars in the TEM images represent 20 nm.

AuNPs were prepared from pentanethiol (C5) capped AuNPs following reported procedures.^40^ Briefly, C5-capped AuNPs were first generated using the Brust–Schiffrin two-phase method.^41^ The C5-capped AuNP and the functionalized thiols were combined (AuNP/thiol = 1:3) with a suitable solvent (CH_2_Cl_2_/MeOH = 1:1) to conduct the ligand exchange reaction. After the reaction was carried out for 3 days, the mixture was purged with N_2_ to remove any trace of solvent. The solid AuNP residue was washed with hexane and diethyl ether and redissolved in D_2_O. The redissolved AuNPs were centrifuged five times with a filter (3 kDa) to remove any excess ligand. As expected, dynamic light scattering (DLS) measurements showed a hydrodynamic diameter of ∼10 nm with no aggregation observed (Figure 2a and Table S1). The spherical particle structure was confirmed via transmission electron microscopy (TEM, Figure 2b).

### 2.2 Headgroup Effects on AuNP Ligand Mobility with Different Headgroups

**Figure 3:**
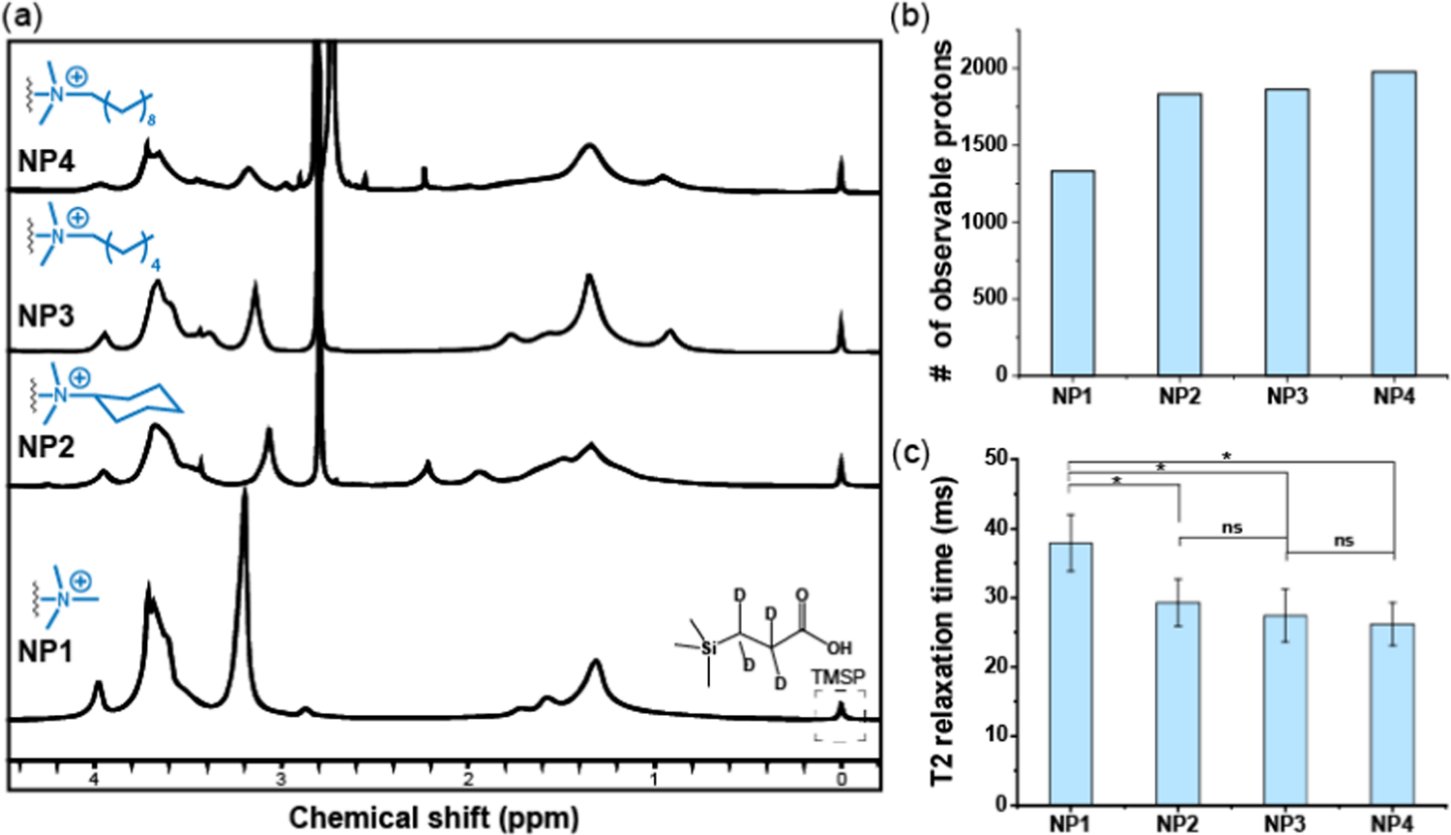
(a) ^1^H NMR spectra of AuNPs in D_2_O at room temperature. The concentration of each AuNP NMR sample is 20 μM. Deuterated trimethylsilyl propanoic acid (TMSP) (140 μM) was used as an internal reference, exhibiting a singlet at 0 ppm. The analysis in (b, c) focused on the 0.5–2 ppm range in the ^1^H NMR spectra, including (b) the quantification of observable protons within this specific range and (c) the measurement of T2 relaxation time for the C11 chains on AuNPs. * = *p*-value <0.05.

^1^H NMR spectroscopy was used to probe the ligand conformational mobility of the AuNPs. As shown in Figure 3a, AuNPs demonstrated substantial broadening of the proton signals compared with the free ligands (Figures S1–S4). Importantly, two broadened proton signals can be easily differentiated: a broad peak from ca. 2.5–4 ppm corresponding to headgroup and TEG protons and a broader NMR resonance at 0.5–2 ppm corresponding to the nonsubstituted portion of the C11-bridged alkyl chain. We focused on the latter C11 chain signals since they do not overlap with other proton signals.

The observed broadening of the proton signal for ligands attached to the AuNP surface compared to free ligands is attributed to alterations in T2 relaxation due to the process wherein nuclear spins lose their initial phase coherence (synchronization) in the transverse plane (perpendicular to the external magnetic field) due to magnetic field inhomogeneities and spin–spin interactions.^42^ Rapidly tumbling molecules experience averaged-out magnetic dipole–dipole interactions between neighboring nuclear spins, reducing their influence on T2 relaxation. In contrast, less mobile environments, such as ligands bound to AuNPs, do not effectively average these interactions, resulting in stronger magnetic field inhomogeneities. Consequently, a faster loss of phase coherence among spins is observed, leading to shorter T2 relaxation times. As T2 is inversely proportional to the peak width, broader peaks were observed for ligands on AuNPs compared to their free forms.

Quantitative NMR was employed to determine the number of observable protons. Deuterated trimethylsilyl propanoic acid (TMSP) was utilized as an internal ref (43). The concentration ratio between TMSP and AuNP was maintained at 7:1. Thus, the number of observable protons on the AuNP ligands was calculated by using the following formula: Observed NP proton numbers = integration value × 7 × 9 (Figure 3b).

Increasing the hydrophobicity of the surface moiety resulted in lower conformational mobility of the ligands, as evidenced by the decreasing T2 values in Figure 3c. This observation can be attributed to the enhanced hydrophobic chain packing facilitated by a more hydrophobic headgroup on the ligand. Notably, the bound ligands on NP1 showed a significantly higher T2 value than those on other AuNPs (*p*-value <0.05). In contrast, only a small difference in T2 values was observed among bound ligands on NP2, NP3, and NP4. A potential explanation for this observation is that hydrophobic groups can form a highly organized arrangement, rendering the system less sensitive to variations in hydrophobicity.

### 2.3 Dynamics of AuNP Ligands at Varying Ionic Strengths and Temperatures

**Figure 4:**
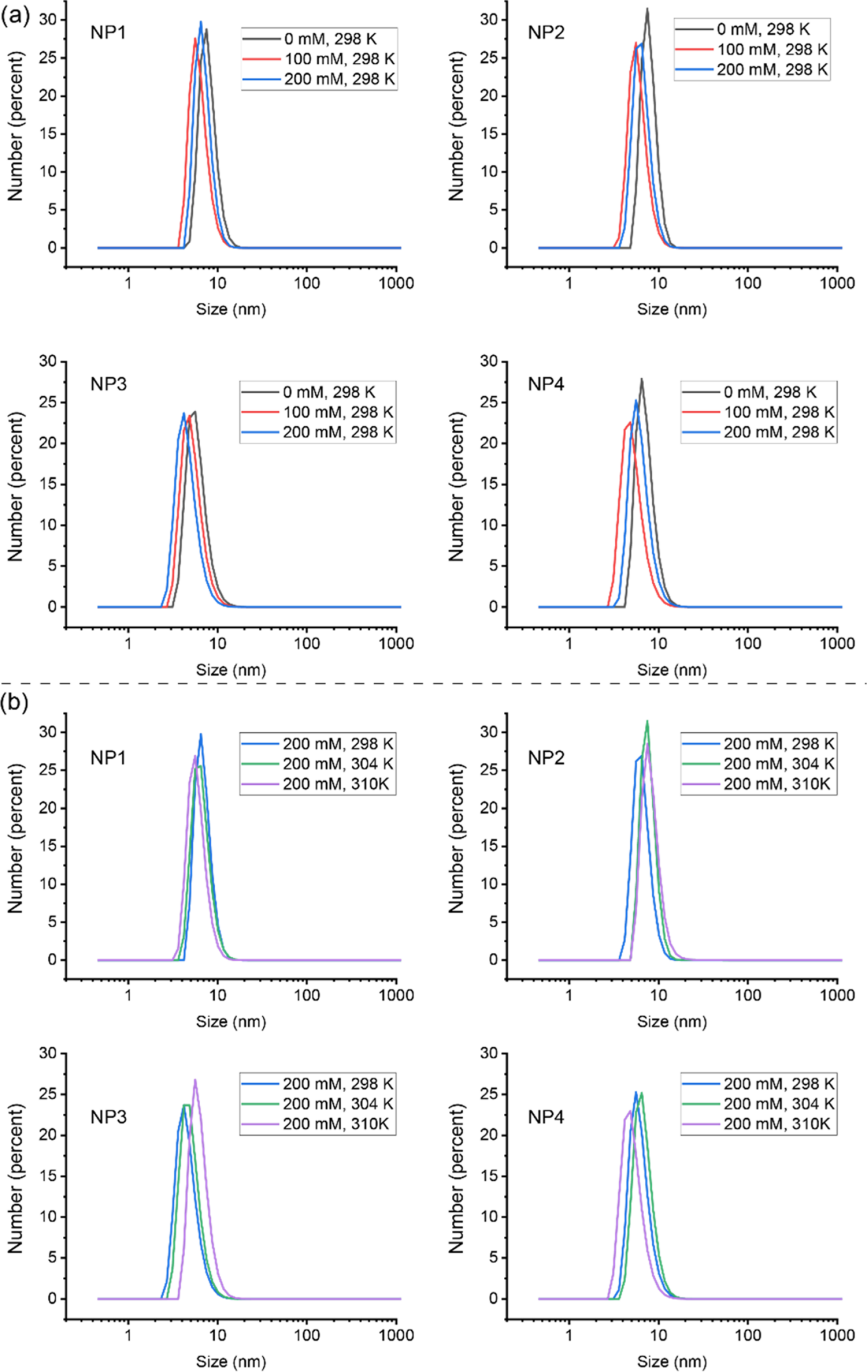
Size distribution of different AuNPs under (a) different NaCl concentrations (0, 100, and 200 mM) and (b) different temperatures (25, 31, and 37 °C). AuNPs displayed excellent stability under all conditions, as evidenced by the absence of aggregation.

We subsequently explored the impact of temperature and ionic strength on the dynamics of bound ligands to gain further insights into the AuNP behavior under diverse environmental conditions. First, the colloidal stability of AuNPs was examined over a range of ionic strengths and temperatures relevant to this study. The ionic strength in AuNP solutions was adjusted by altering NaCl concentrations (0, 100, and 200 mM). The AuNPs in 200 mM NaCl solutions were then divided into three groups and incubated at 25 °C (298 K), 31 °C (304 K), and 37 °C (310 K), respectively, for 10 min. DLS measurements were used to determine the stability of the samples described above. As shown in Figure 4a,b, there was no significant size change at the various NaCl concentrations and temperatures, demonstrating the high stability of the AuNPs.

**Figure 5:**
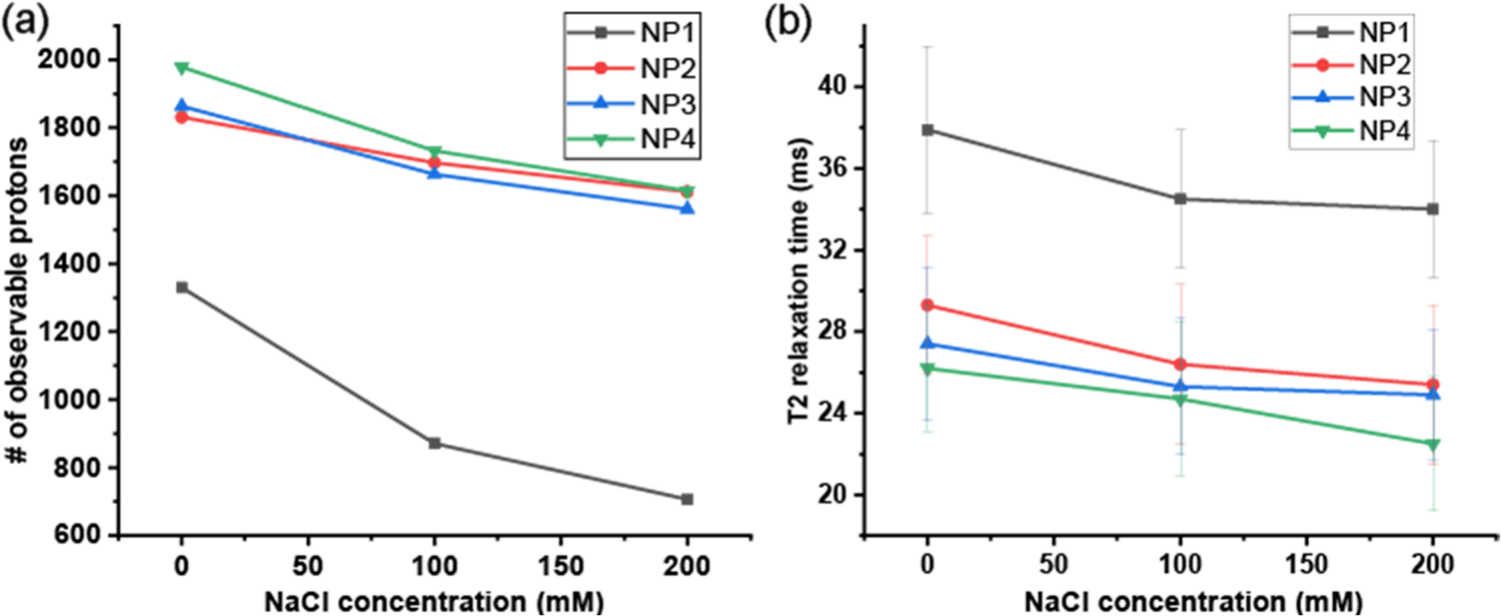
Quantitative NMR and T2 relaxation measurement in D_2_O of the C11 chains on AuNPs at different NaCl concentrations. The AuNP concentration of each NMR sample is 20 μM. (a) The number of observable protons in ^1^H NMR spectra. TMSP was used as an internal reference (140 μM). (b) T2 relaxation was measured by introducing the CPMG pulse sequence.^44^

We used quantitative NMR and T2 measurements to analyze the C11 chain of AuNPs at different ionic strengths. As depicted in Figure 5a, increasing the NaCl concentration for all four AuNPs led to the loss of proton signals in the ^1^H NMR spectrum. Moreover, bound ligands at higher salt concentrations exhibited shorter T2 relaxation times (Figure 5b). The decrease in the proton integrals and T2 values at elevated salt concentrations can be attributed to ligand conformational changes. With increased ionic strength, ligand headgroups experience weaker electrostatic repulsion, resulting in more compact chain packing. Additionally, at higher salt concentrations, the hydrophobic moieties on the ligand will tend to aggregate due to the “salting out” effect, leading to even tighter packing. Finally, hydrophobic terminal groups can facilitate chain packing by attracting van der Waals forces and hydrophobic interactions. The surface-bound ligand with more hydrophobic headgroups (NP4) at the same NaCl concentration had shorter T2, indicating less conformational mobility (Figure 5b).

**Figure 6:**
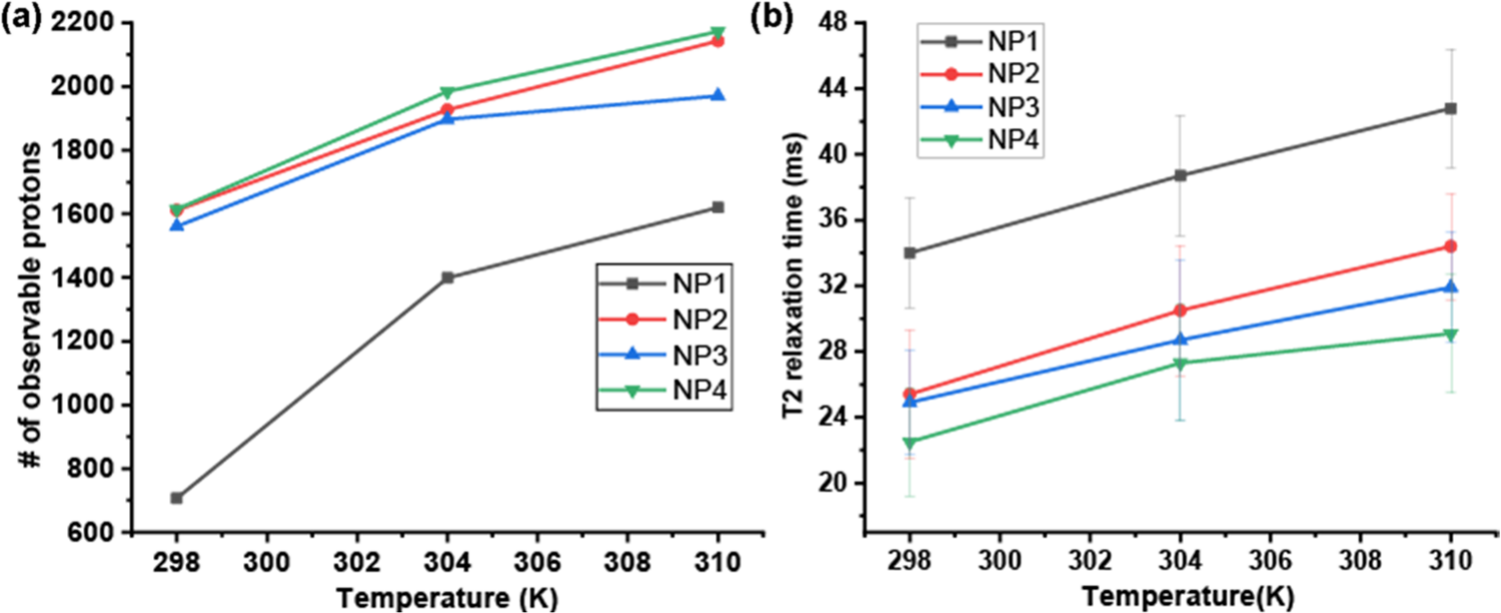
C11 chains on AuNPs were studied at different temperatures via quantitative NMR and T2 relaxation measurement in D_2_O. The concentration of each NMR sample is 20 μM. (a) The number of observable protons in ^1^H NMR spectra. TMSP was used as an internal reference (140 μM). (b) T2 relaxation was measured by introducing the CPMG pulse sequence.

After investigating the effects of ionic strength on ligand conformation, we examined the impact of temperature on the conformation of surface-bound ligands using quantitative NMR integration and T2 measurements. The AuNPs in 200 mM NaCl solutions were heated to either 31 °C (304 K) or 37 °C (310 K) in the NMR cavity and equilibrated for 10 min before taking any measurements. As shown in Figure 6a, the proton signal loss induced by the high NaCl concentration recovered with increasing temperature. Ligands at higher temperatures exhibited longer T2, consistent with the increase in peak sharpness in the quantitative NMR results (Figure 6b). Additionally, ligands with greater hydrophobicity in their terminal groups exhibited shorter T2 (lower mobility) at all temperatures, following the same trend observed in the study of AuNP ligands with different ionic strengths.

The increased mobility of surface ligands on AuNPs at higher temperatures can be explained by several factors. First, the increase in temperature leads to enhanced molecular motion, resulting in greater ligand mobility and more disordered conformations. Second, hydrophobic interactions are inherently entropically driven and diminished at increased temperatures, allowing the ligands to become more mobile.^45^

## 3 Conclusions

The dynamics of the monolayer ligands attached to AuNPs are governed by both entropic and enthalpic effects.^46^ The long hydrophobic segments interact through attractive van der Waals forces, while the ammonium headgroups generate electrostatic repulsion. Moreover, the water molecules surrounding the headgroups and trapped between the TEG linkers contribute to the packing of the C11 chain through hydrophobic interactions. Ultimately, the degree of monolayer compaction of AuNPs depends on the balance among these opposing forces. We have used solution NMR spectroscopy to determine these dynamics of AuNP ligands in situ, focusing on the impact of headgroup structure on monolayer packing. These studies show that increasing end-group hydrophobicity favors ligand packing, reducing mobility. Increases in ionic strength amplify this effect, while increasing the temperature diminishes packing. These studies show that subtle changes in the headgroup structure can dramatically alter the ligand mobility and that NMR provides a tool for characterizing this mobility. The degree of ligand organization is a key driver in the interactions of nanoparticles with the environment and biosystems. The integration of synthetic and analytical methods provides a foundational tool for nanoparticle development for a wide range of biomedical and chemical applications, such as the development of nanobased sensing and drug delivery platforms.

## 4 Methods

### 4.1 Gold Nanoparticle (AuNP) Synthesis

The 2 nm gold core and AuNPs were prepared following reported procedures. The final concentration of nanoparticles dispersed in water was measured by ultraviolet (UV) spectroscopy on a Molecular Devices SpectraMax M2 instrument at 506 nm.

### 4.2 Dynamic Light Scattering (DLS) and Transmission Electron Microscopy (TEM)

Hydrodynamic diameter of the AuNPs (1 μM) was measured by DLS using a Malvern Zetasizer Nano ZS instrument. The measurement angle was 173° (backscatter). Data were analyzed by the “multiple narrow modes” (high resolution) based on non-negative least squares (NNLS).

TEM samples of AuNPs were prepared by placing one drop of the desired solution (1 μM) onto a 300-mesh Cu grid-coated with a carbon film. These samples were analyzed and photographed using JEOL CX-100 electron microscopy. The respective sizes, standard deviations, and PDI values can be found in the Supporting Information (Table S1).

### 4.3 ^1^H NMR Spectra of AuNPs at Different Conditions

^1^H NMR spectra were obtained by using a Bruker Advance III 400 MHz NMR device. TMSP (140 μM) was used as an internal standard for obtaining the number of protons. Briefly, gold nanoparticles were dispersed at a concentration of 20 μM. The area between approximately 0.5 and 2 ppm was selected and fine-tuned to cover the entire area under the peaks before integration. The respective ^1^H NMR spectra can be found in the Supporting Information (Figures S5–S24).

## Supporting Information Available

AuNP synthesis; methods of DLS and TEM; ^1^H NMR spectra of free ligands for AuNPs; and ^1^H NMR spectra of AuNPs at different conditions (PDF)
