# Supplementary material for: Modulation of Gold Nanoparticle Ligand Structure–Dynamic Relationships Probed Using Solution NMR
Source: ACS Nanosci Au. 2023 Nov 8;4(1):62–8. doi: 10.1021/acsnanoscienceau.3c00042 (PMC10885325; doi:10.1021/acsnanoscienceau.3c00042)
Supplement: Supplementary file 1 — ng3c00042_si_001.pdf [file ng3c00042_si_001.pdf]

Electronic supplementary information

Modulation of Gold Nanoparticle Ligand Structure-Dynamic Relationships Probed using Solution NMR

Rui Huang, Stefano Fedeli, Cristina-Maria Hirschbiegel, Xianzhi Zhang and Vincent M. Rotello*

Department of Chemistry, University of Massachusetts Amherst, 710 North Pleasant Street, Amherst, Massachusetts 01003, United States

1. Sizes of AuNPs measured via Dynamic Light Scattering (DLS)

Tab. S1 Sizes of each nanoparticle species with respective standard deviation and PDI measured via DLS.

NP	Size (nm)	St. Dev. (nm)	PDI
NP1	6.461	1.682	0.771
NP2	5.564	1.409	0.170
NP3	6.388	1.717	0.485
NP4	8.729	2.385	0.343

2. ^{1}H NMR spectra of free ligands for AuNPs

^{1}H-NMR spectra were obtained using a Bruker Advance III 400 MHz NMR device.

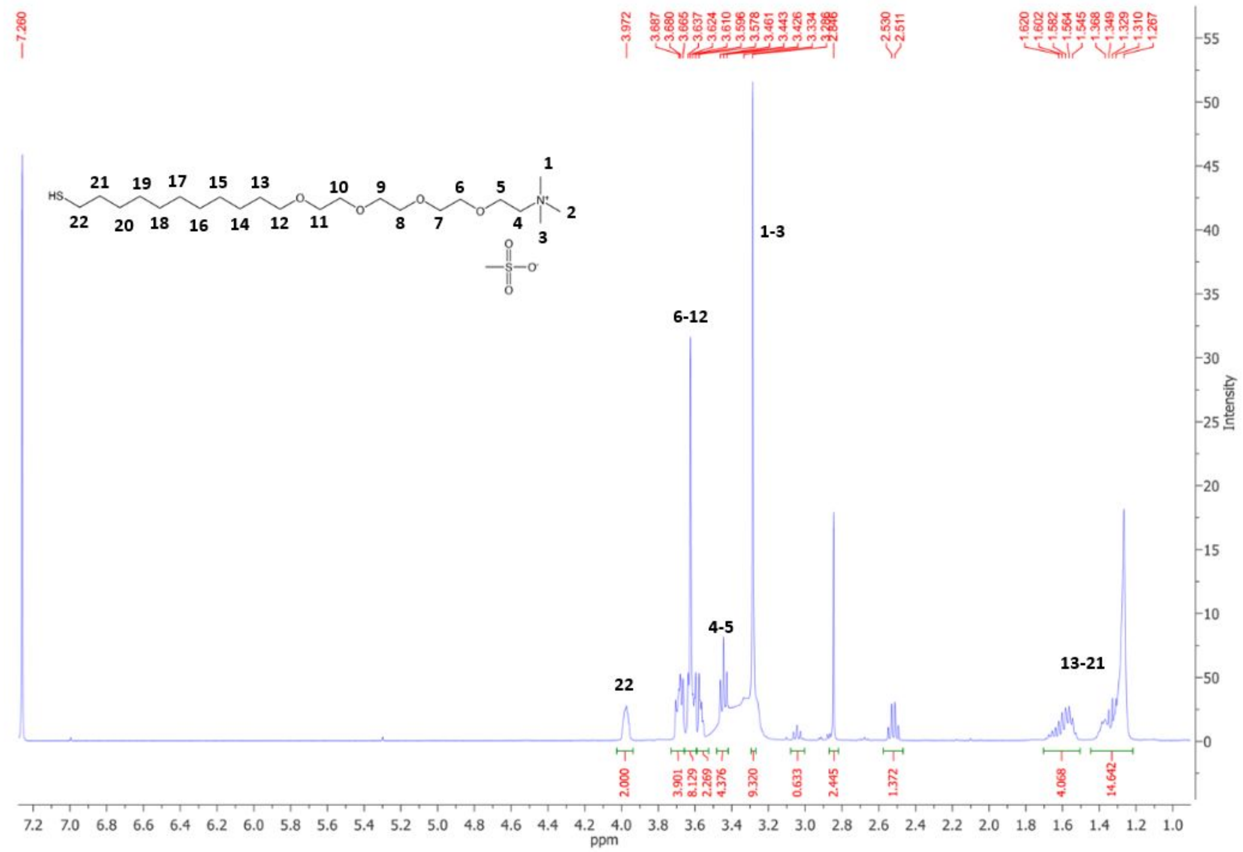

Fig. S1 ^{1}H NMR spectra of free ligands for NP1.

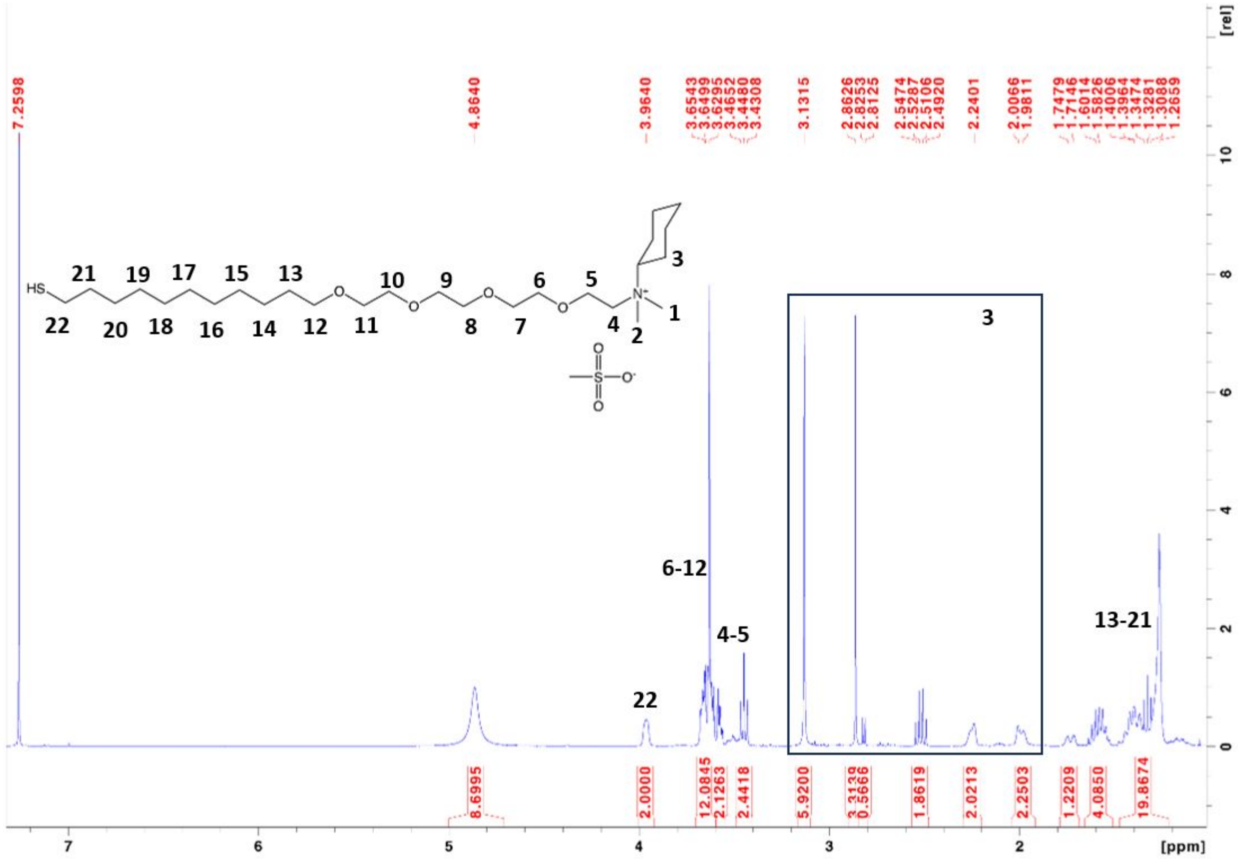

Fig. S2 ^{1}H NMR spectra of free ligands for NP2.

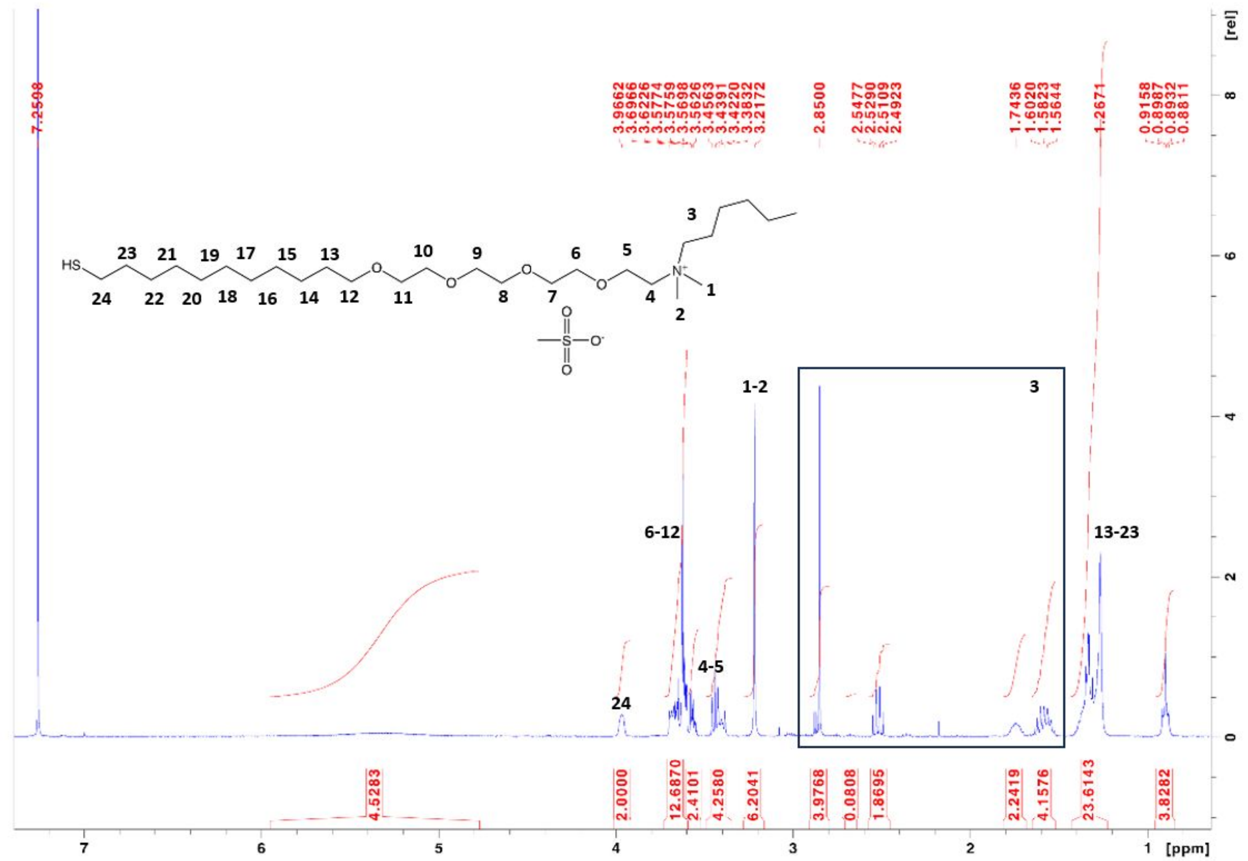

Fig S3. ^{1}H NMR spectra of free ligands for NP3.

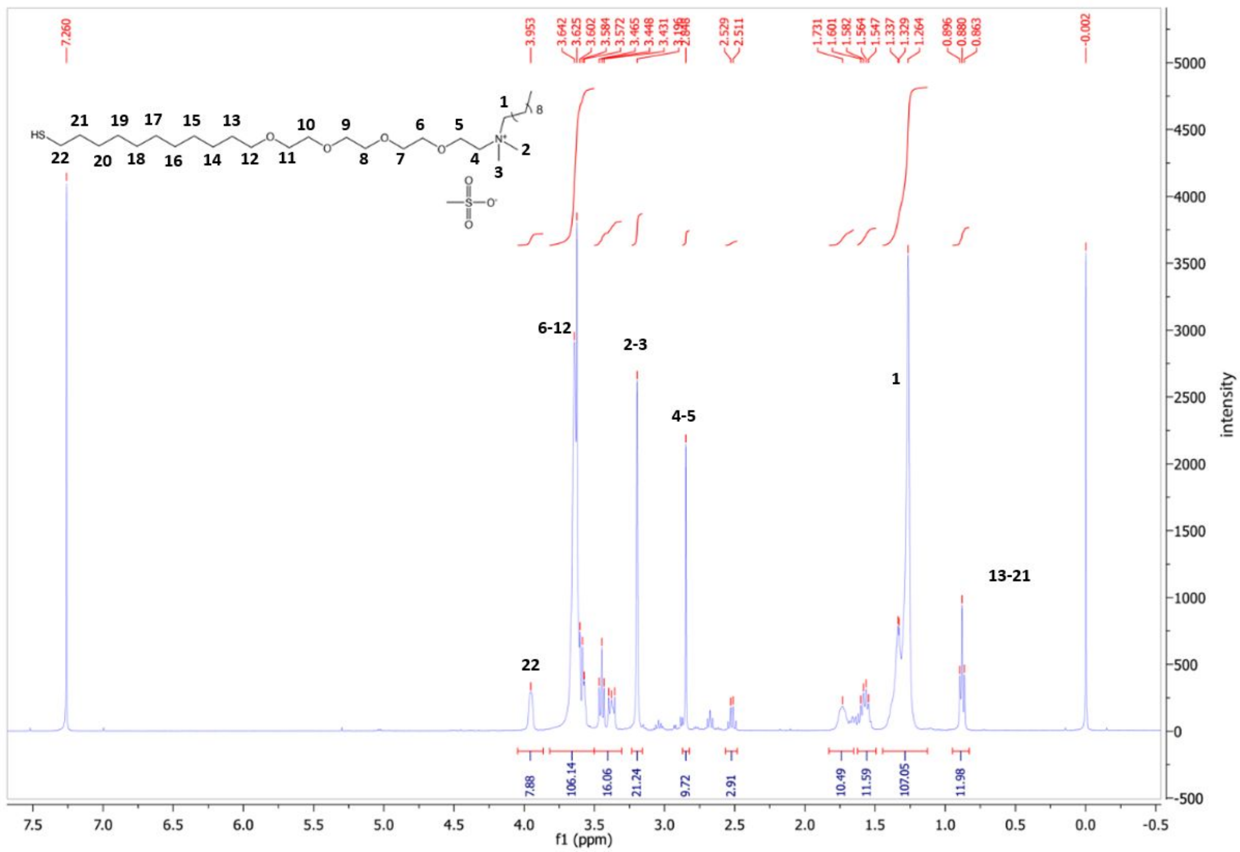

Fig. S4 ^{1}H NMR spectra of free ligands for NP4.

3. ^{1}H NMR spectra of AuNPs at different conditions

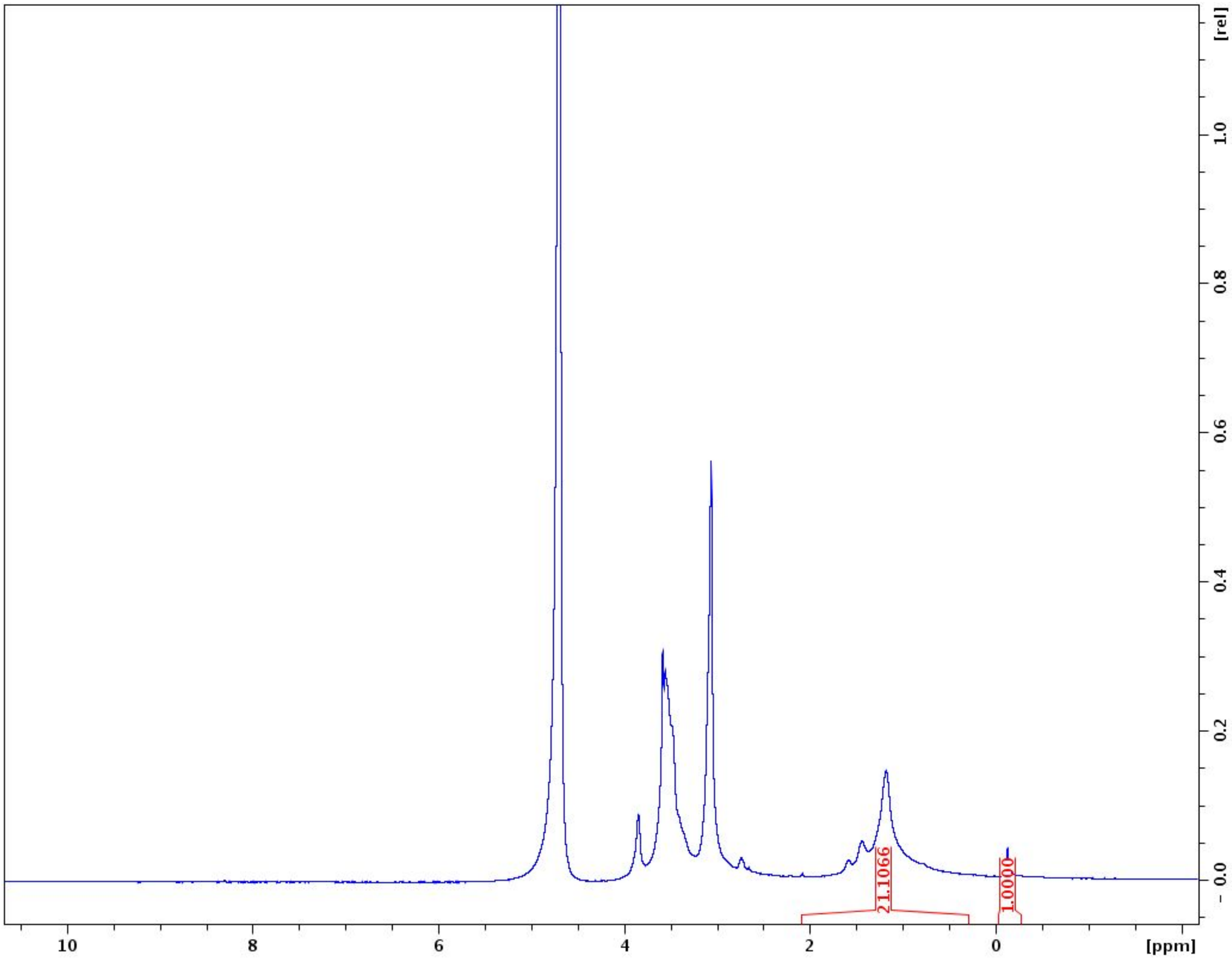

Fig. S5 ^{1}H NMR spectra of NP1 at 0mM NaCl, 298K. Deuterated trimethylsilyl propanoic acid (TMSP) (140 µM) was used as an internal reference, exhibiting a singlet at 0 ppm.

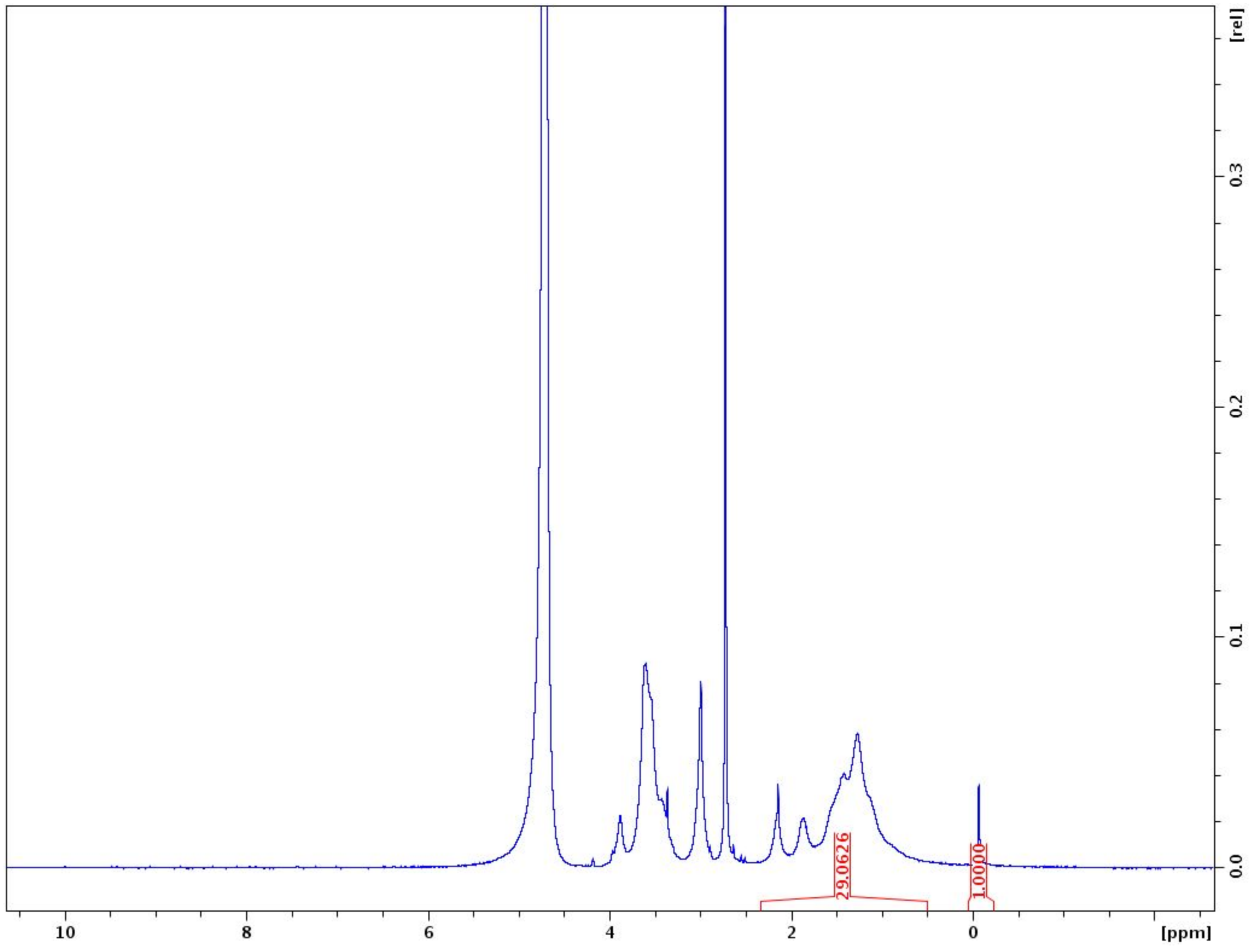

Fig. S6 ^{1}H NMR spectra of NP2 at 0mM NaCl, 298K. Deuterated trimethylsilyl propanoic acid (TMSP) (140 µM) was used as an internal reference, exhibiting a singlet at 0 ppm.

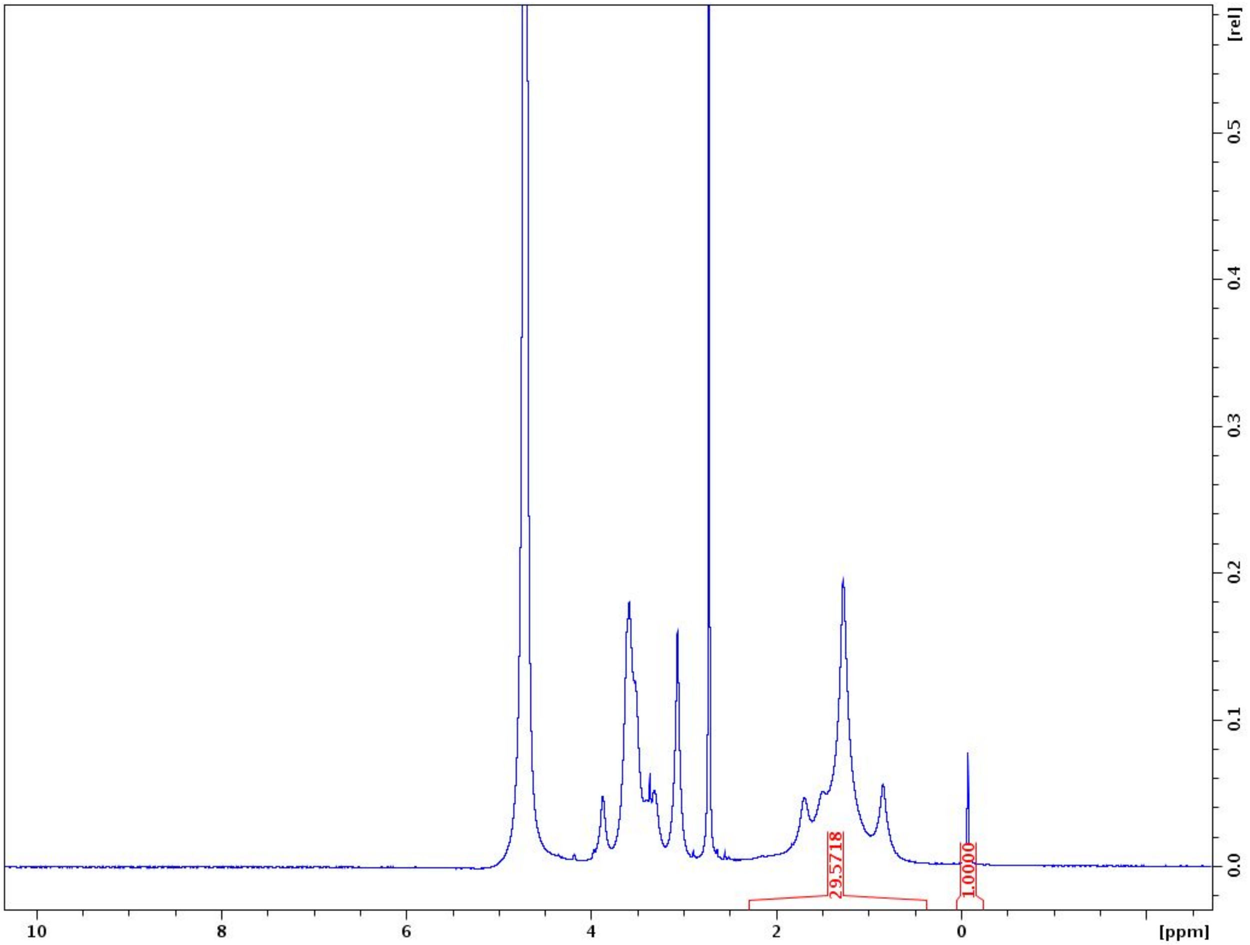

Fig. S7 ^{1}H NMR spectra of NP3 at 0mM NaCl, 298K. Deuterated trimethylsilyl propanoic acid (TMSP) (140 µM) was used as an internal reference, exhibiting a singlet at 0 ppm.

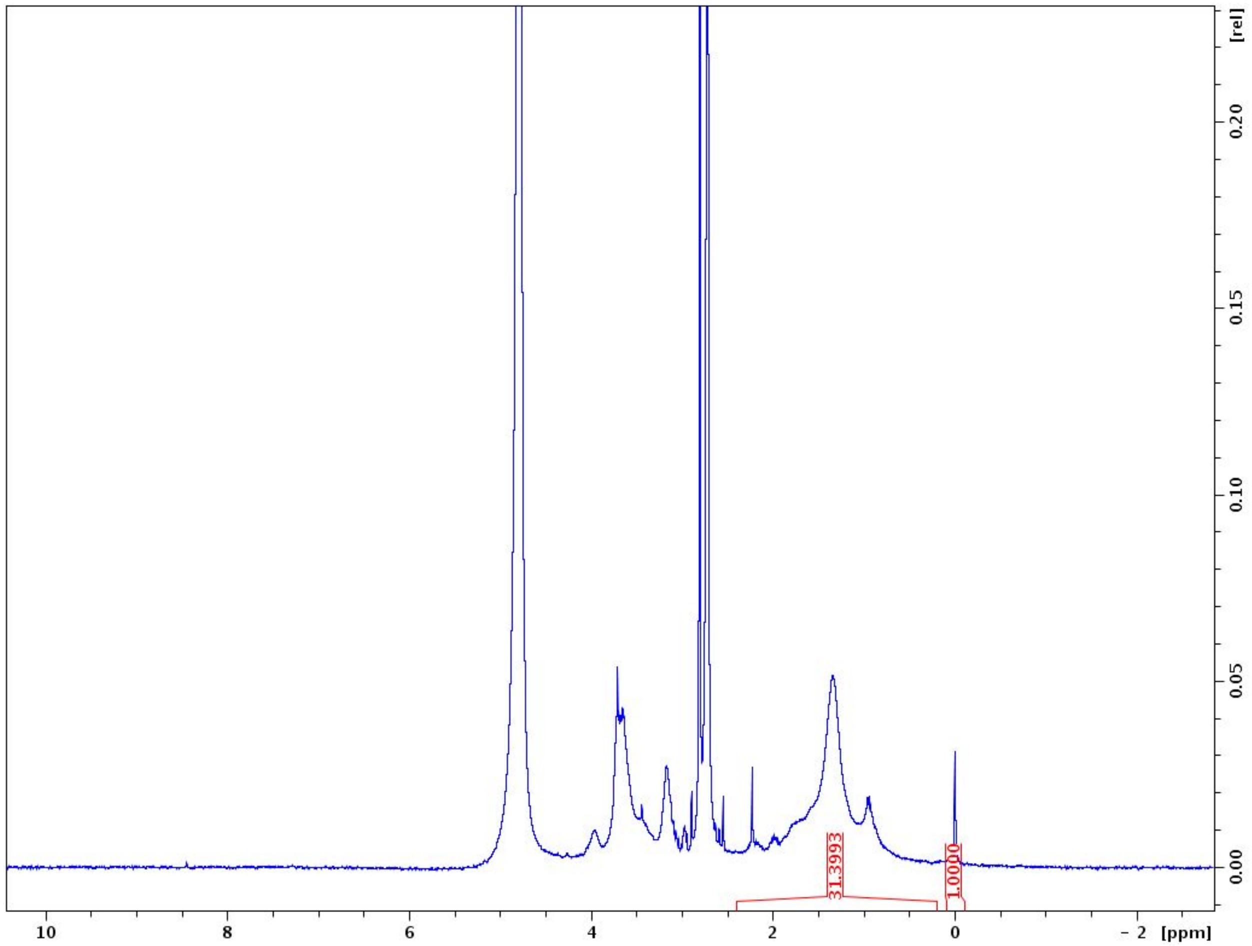

Fig. S8 ^{1}H NMR spectra of NP4 at 0mM NaCl, 298K. Deuterated trimethylsilyl propanoic acid (TMSP) (140 µM) was used as an internal reference, exhibiting a singlet at 0 ppm.

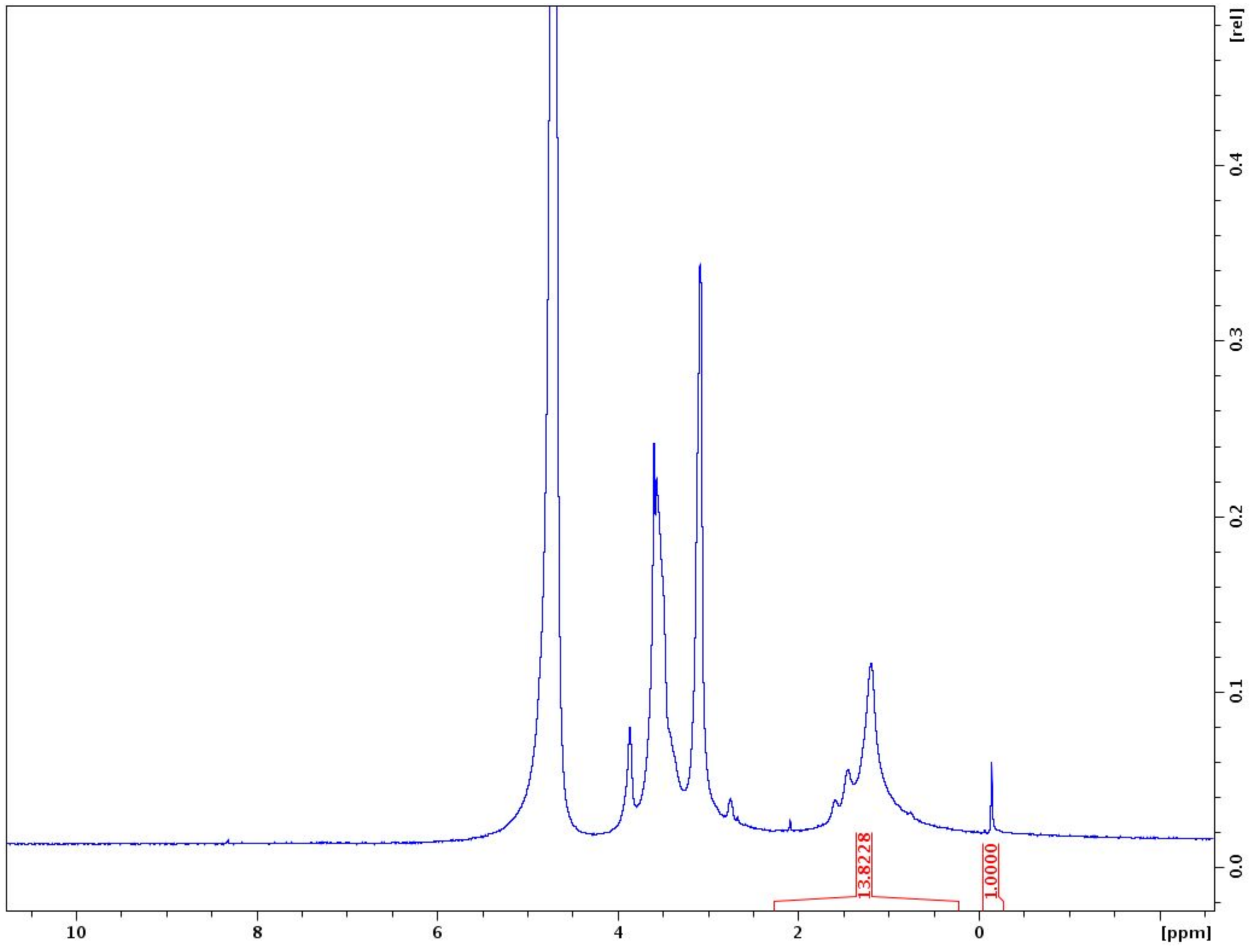

Fig. S9 ^{1}H NMR spectra of NP1 at 100mM NaCl, 298K. Deuterated trimethylsilyl propanoic acid (TMSP) (140 µM) was used as an internal reference, exhibiting a singlet at 0 ppm.

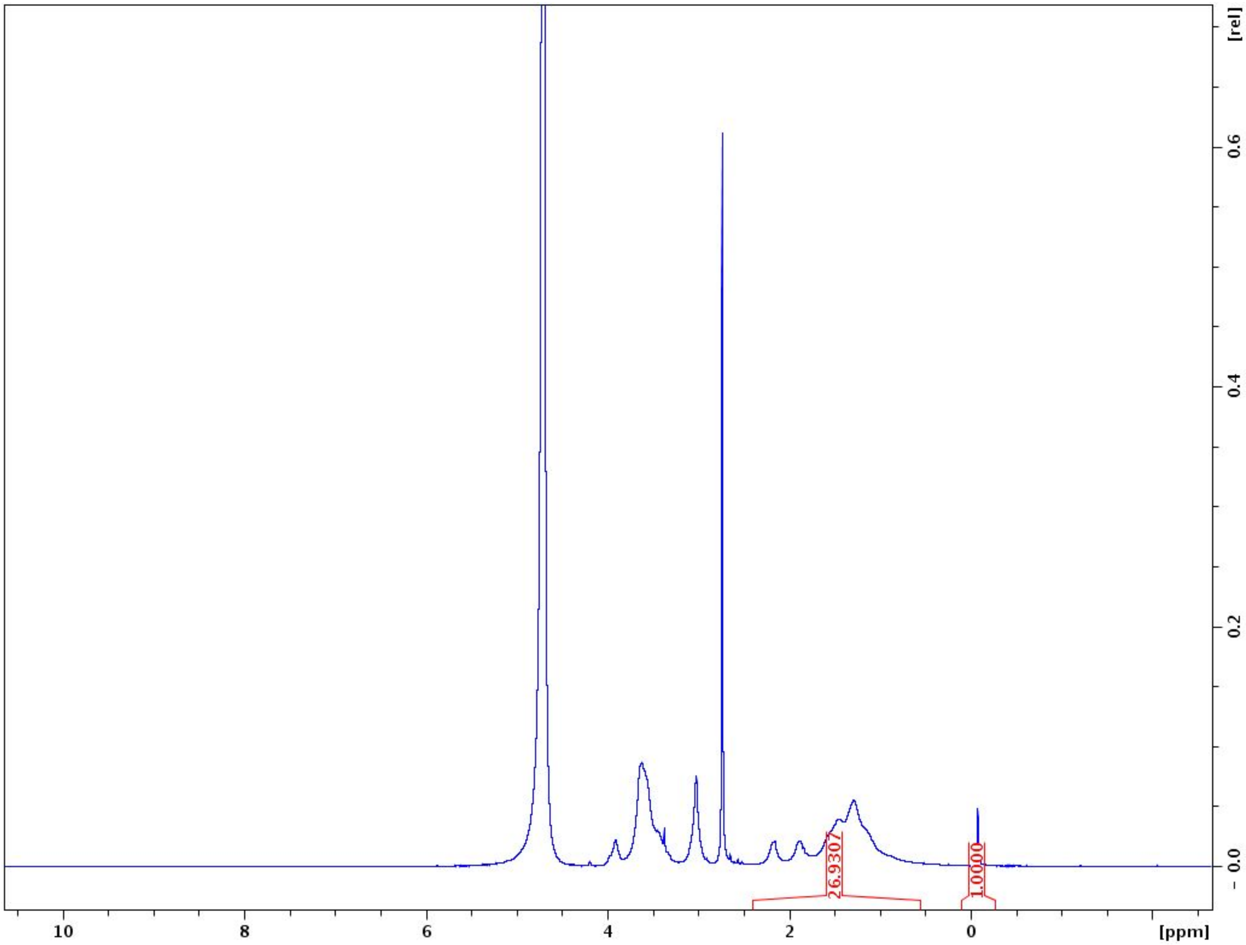

Fig. S10 ^1H NMR spectra of NP2 at 100mM NaCl, 298K. Deuterated trimethylsilyl propanoic acid (TMSP) (140 µM) was used as an internal reference, exhibiting a singlet at 0 ppm.

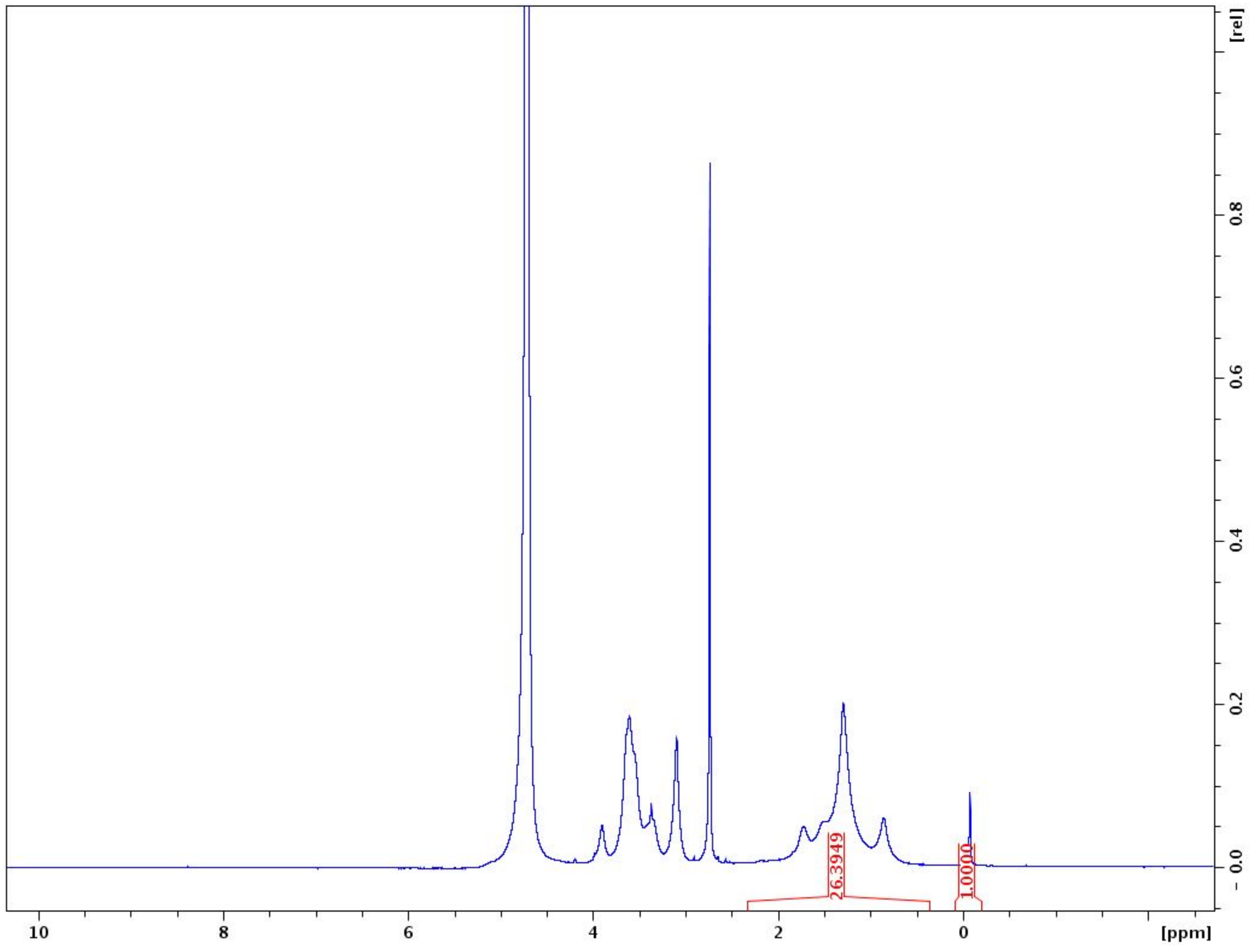

Fig. S11 ^{1}H NMR spectra of NP3 at 100mM NaCl, 298K. Deuterated trimethylsilyl propanoic acid (TMSP) (140 µM) was used as an internal reference, exhibiting a singlet at 0 ppm.

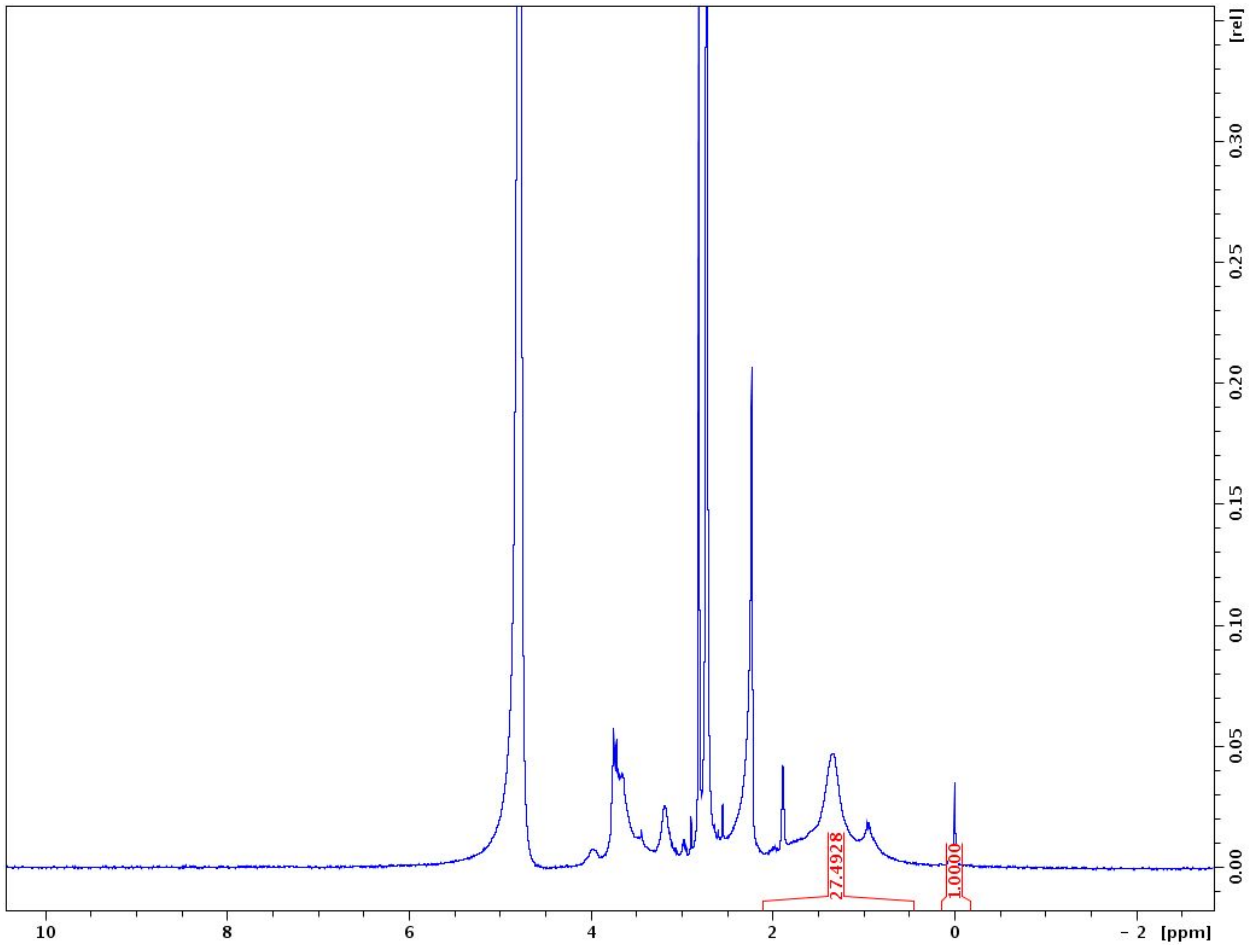

Fig. S12 ^{1}H NMR spectra of NP4 at 100mM NaCl, 298K. Deuterated trimethylsilyl propanoic acid (TMSP) (140 µM) was used as an internal reference, exhibiting a singlet at 0 ppm.

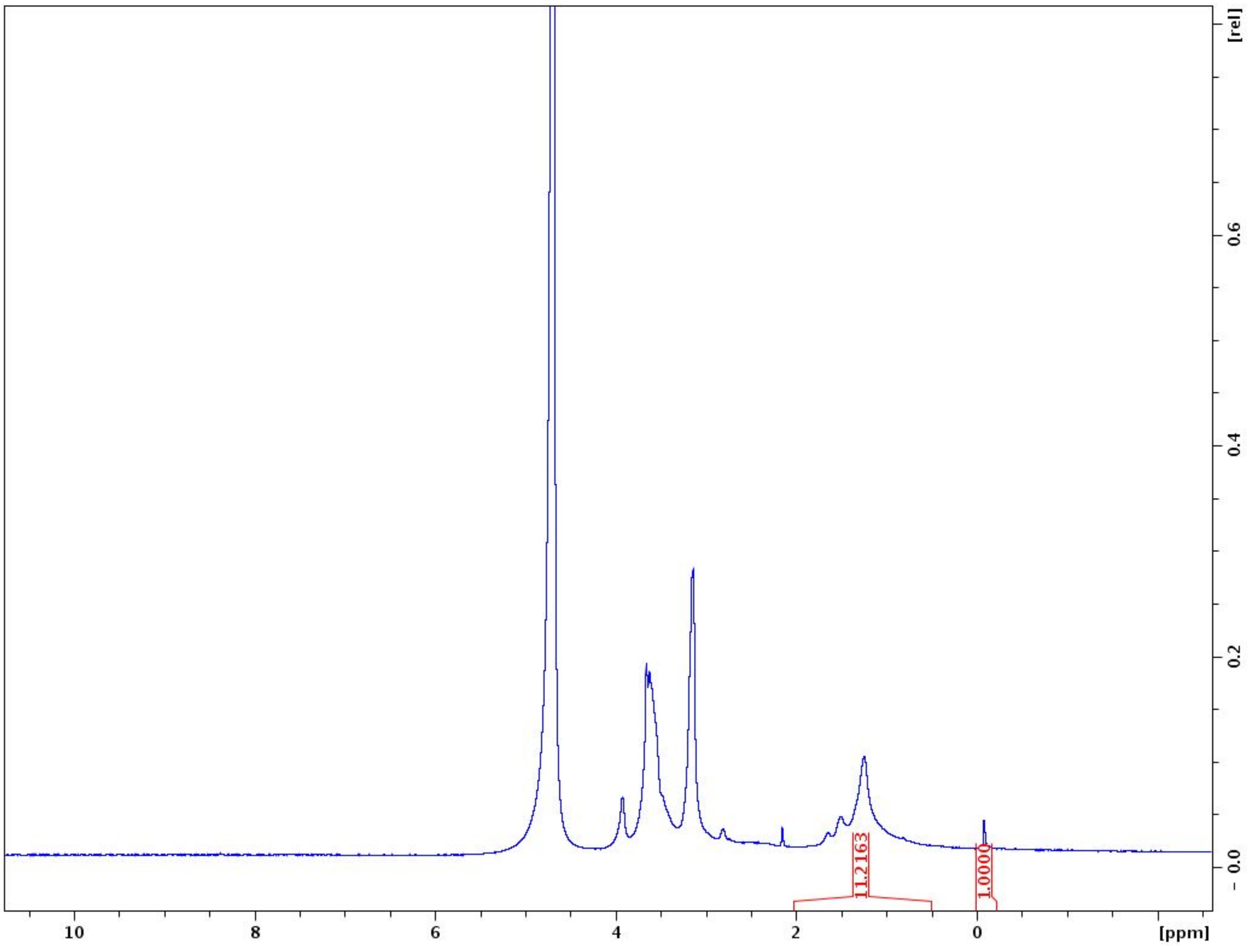

Fig. S13 ^{1}H NMR spectra of NP1 at 200mM NaCl, 298K. Deuterated trimethylsilyl propanoic acid (TMSP) (140 µM) was used as an internal reference, exhibiting a singlet at 0 ppm.

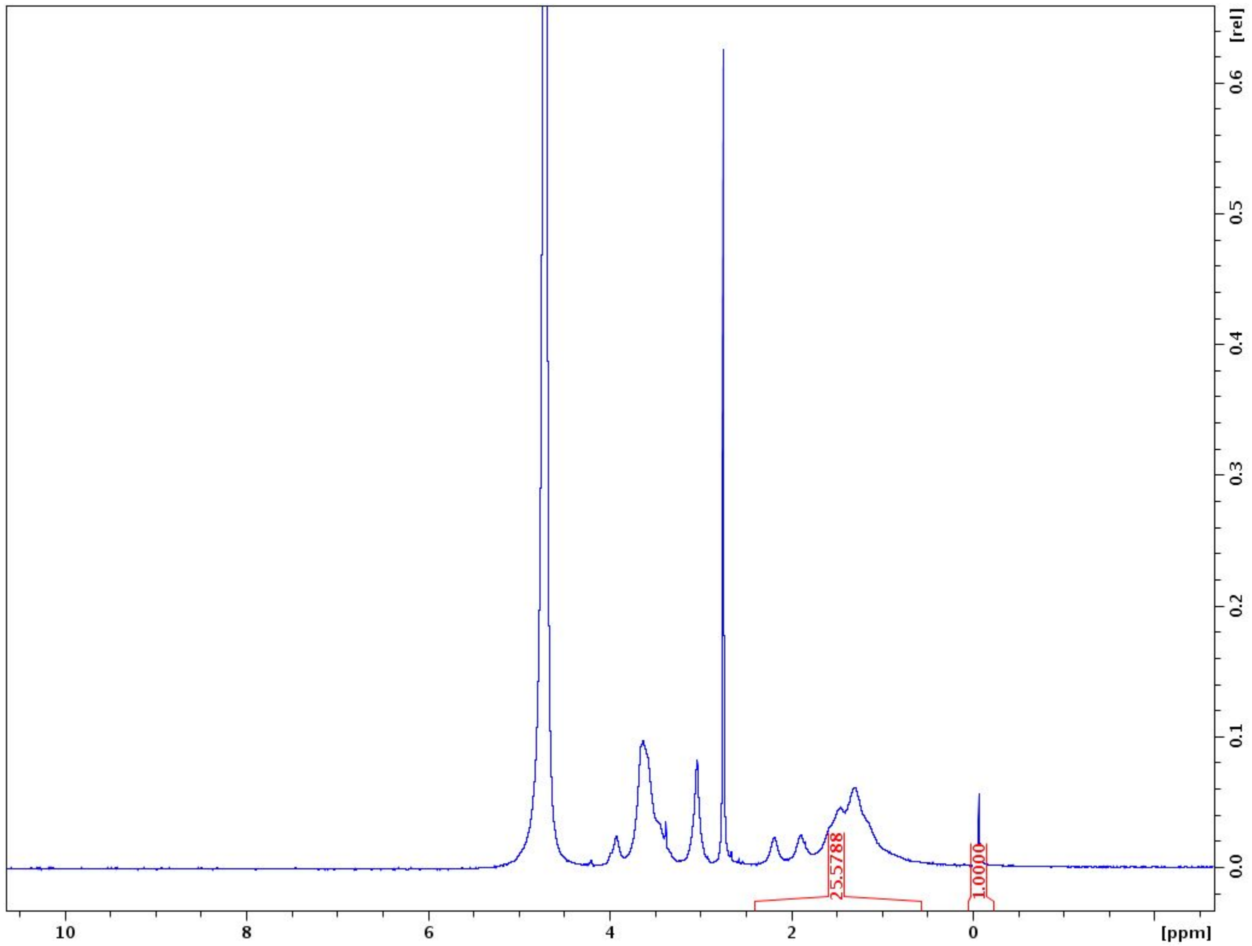

Fig. S14 ^{1}H NMR spectra of NP2 at 200mM NaCl, 298K. Deuterated trimethylsilyl propanoic acid (TMSP) (140 µM) was used as an internal reference, exhibiting a singlet at 0 ppm.

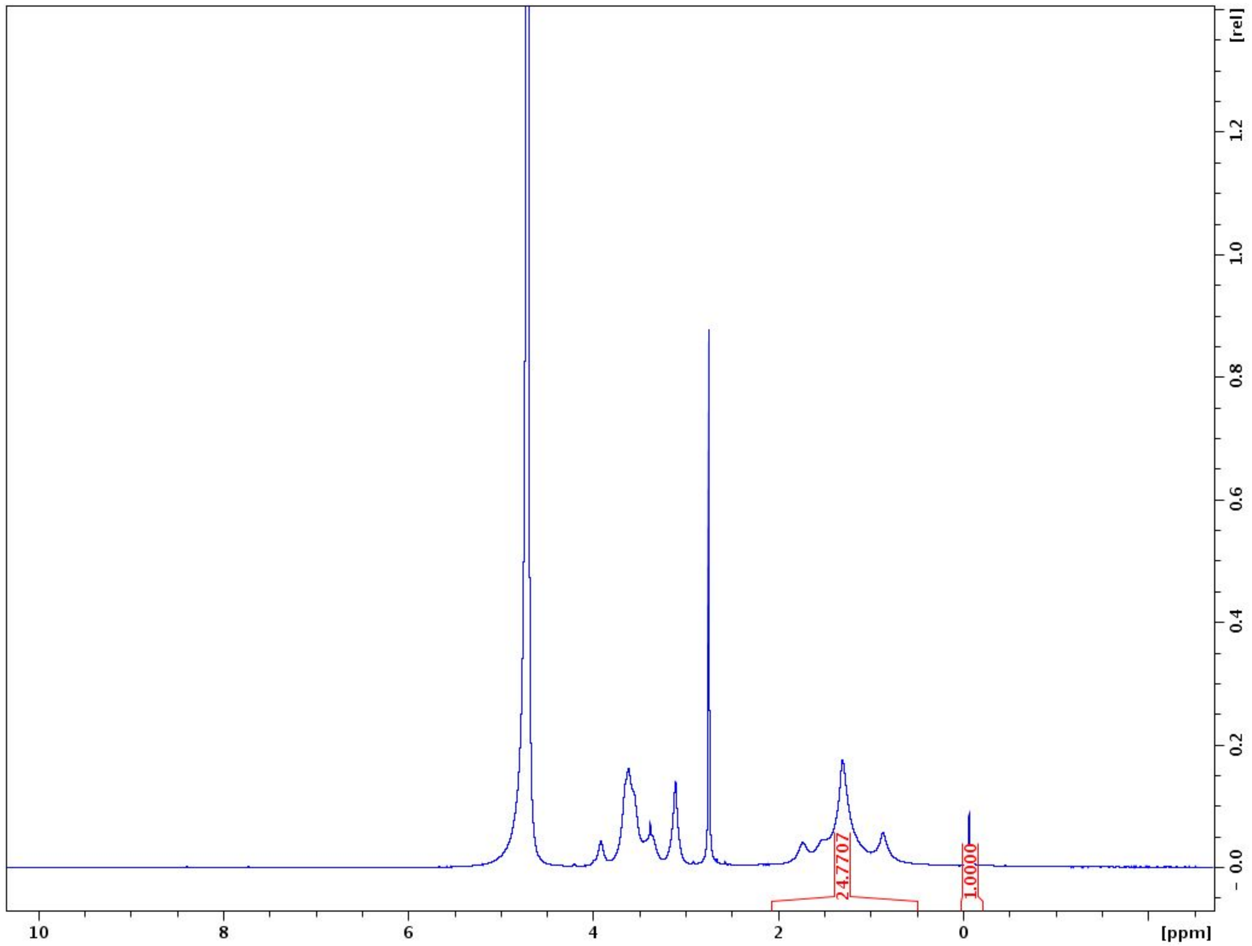

Fig. S15 ^{1}H NMR spectra of NP3 at 200mM NaCl, 298K. Deuterated trimethylsilyl propanoic acid (TMSP) (140 µM) was used as an internal reference, exhibiting a singlet at 0 ppm.

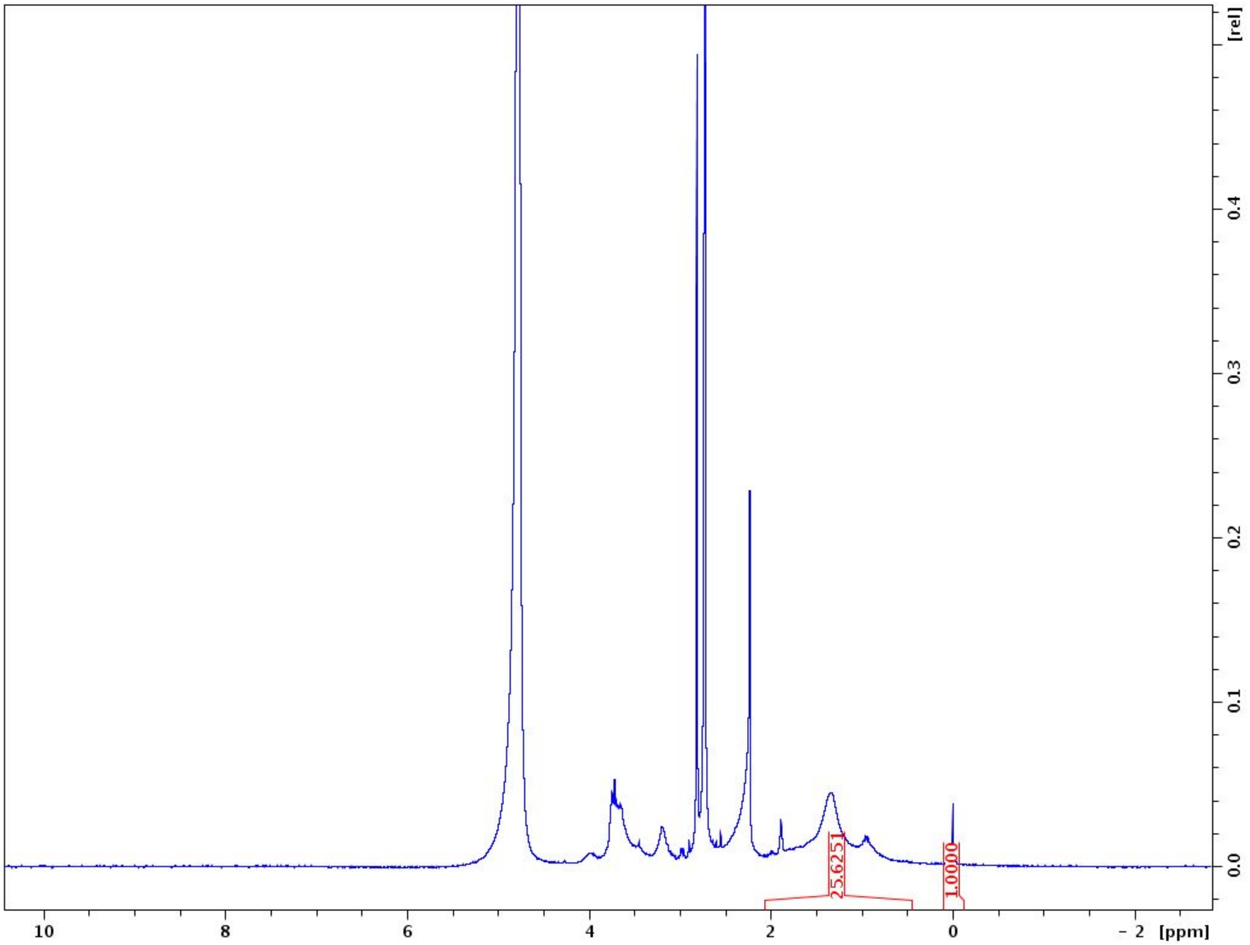

Fig. S16 ^{1}H NMR spectra of NP4 at 200mM NaCl, 298K. Deuterated trimethylsilyl propanoic acid (TMSP) (140 µM) was used as an internal reference, exhibiting a singlet at 0 ppm.

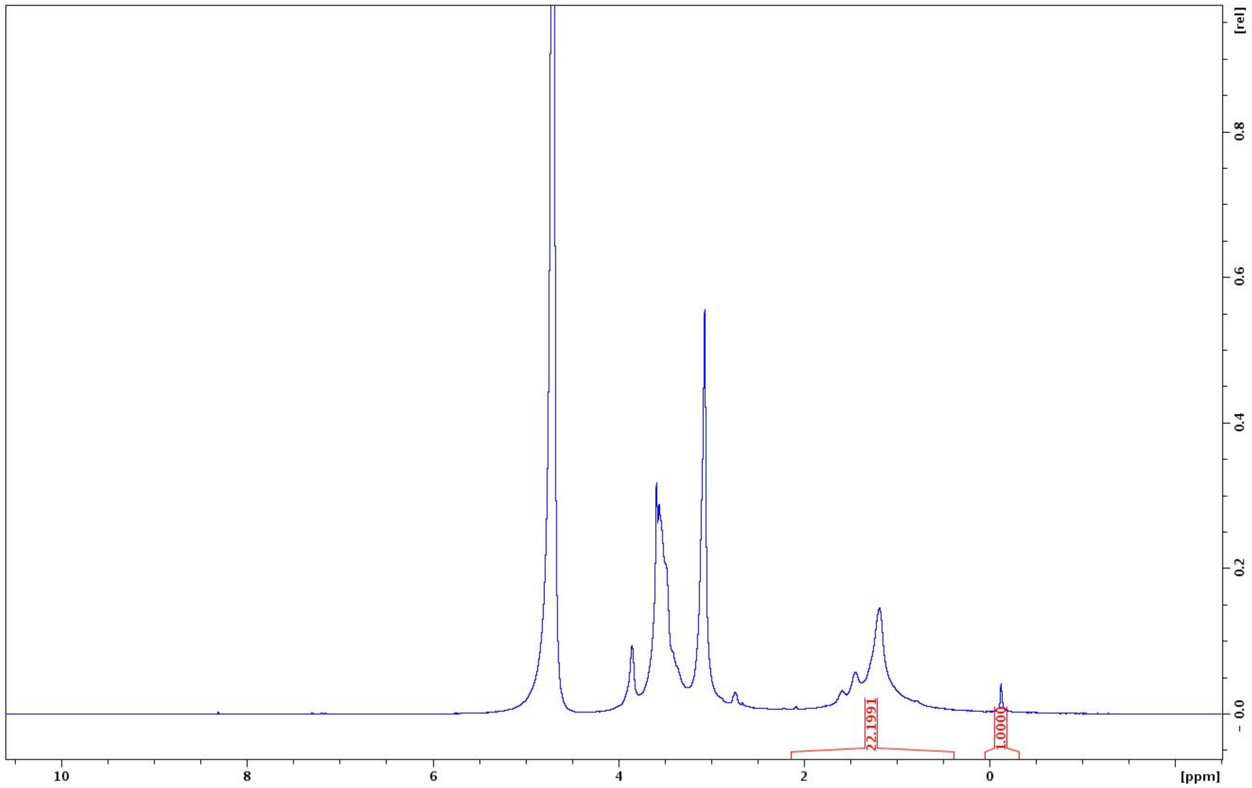

Fig. S17 ^{1}H NMR spectra of NP1 at 200mM NaCl, 304K. Deuterated trimethylsilyl propanoic acid (TMSP) (140 µM) was used as an internal reference, exhibiting a singlet at 0 ppm.

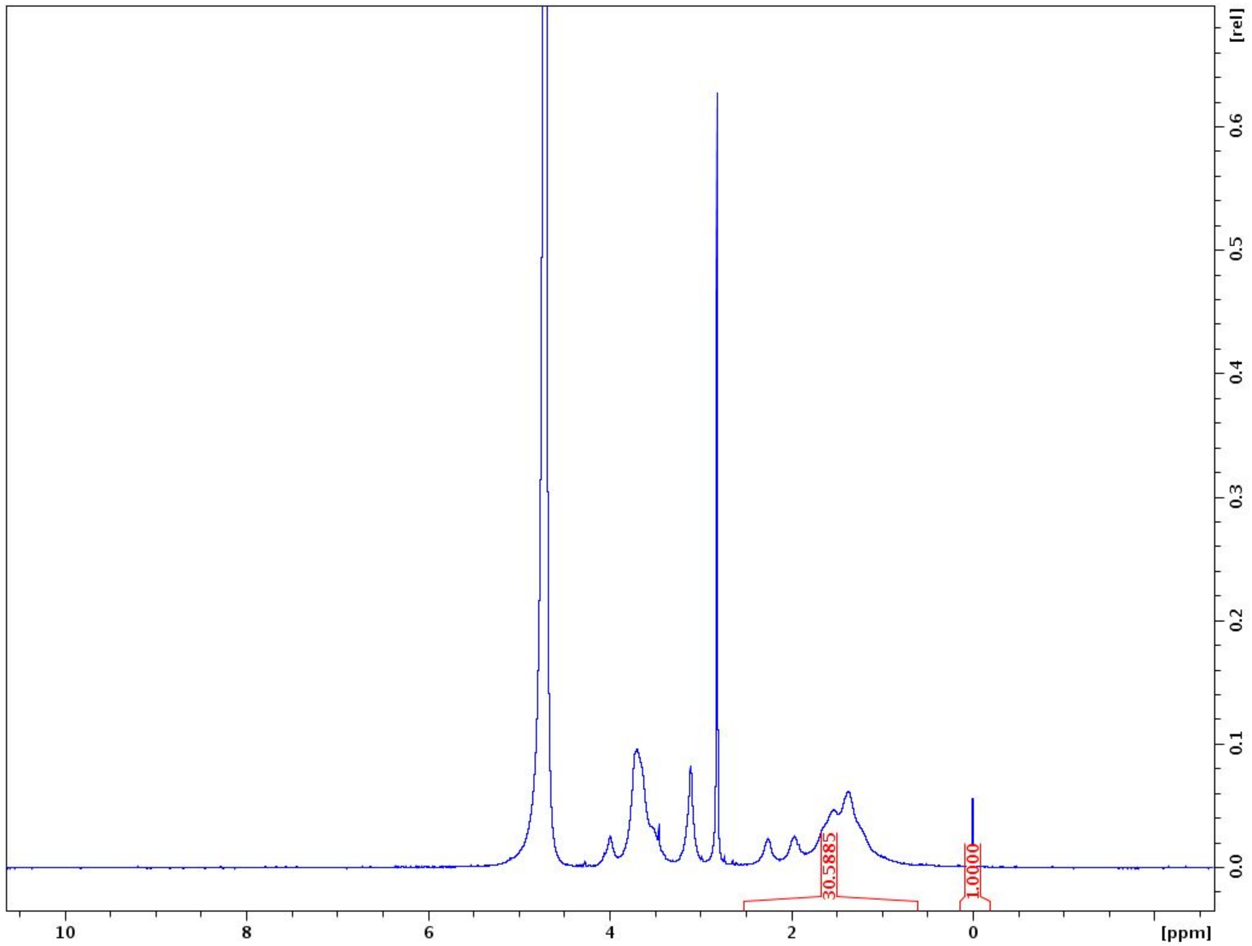

Fig. S18 ^{1}H NMR spectra of NP2 at 200mM NaCl, 304K. Deuterated trimethylsilyl propanoic acid (TMSP) (140 µM) was used as an internal reference, exhibiting a singlet at 0 ppm.

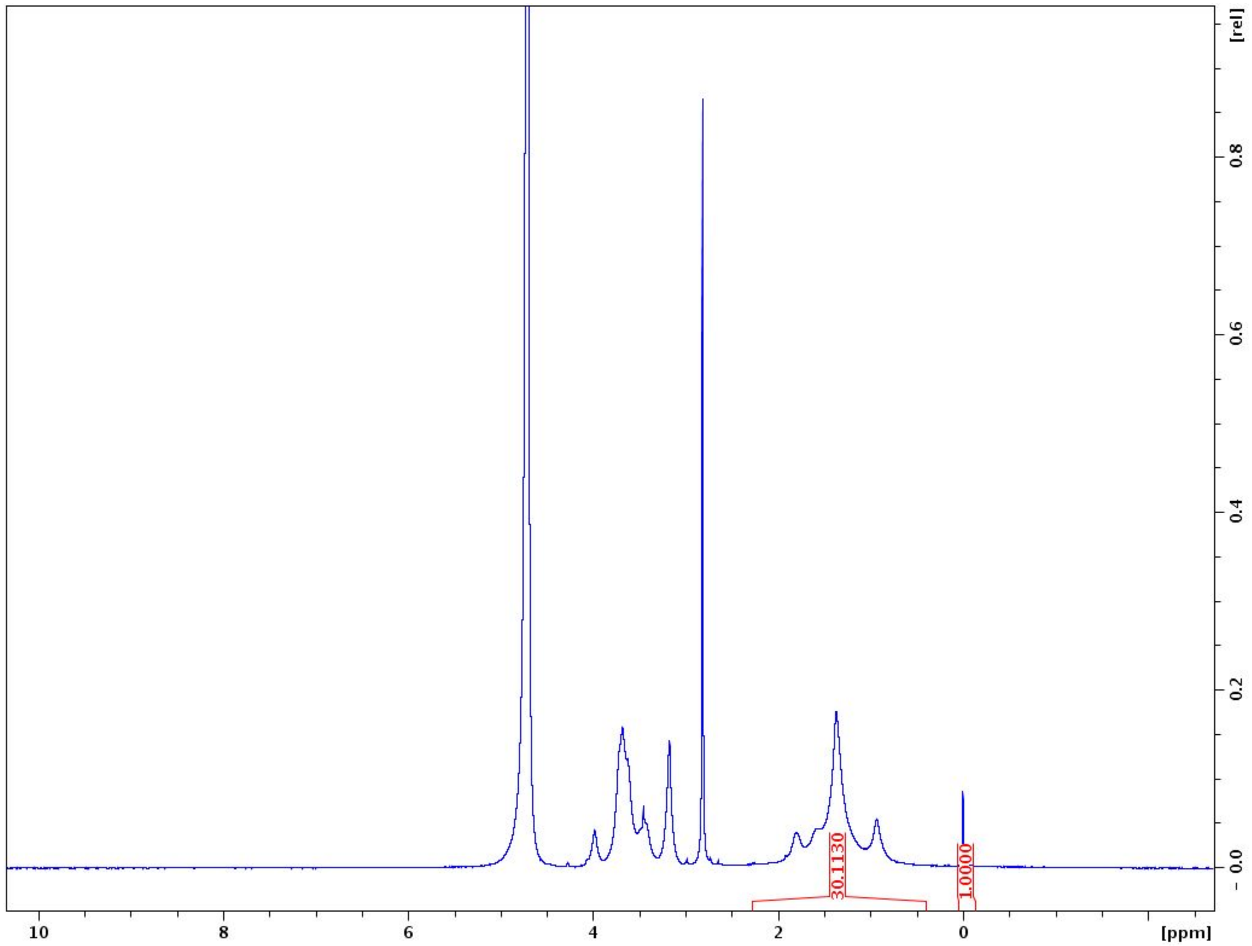

Fig. S19 ^{1}H NMR spectra of NP3 at 200mM NaCl, 304K. Deuterated trimethylsilyl propanoic acid (TMSP) (140 µM) was used as an internal reference, exhibiting a singlet at 0 ppm.

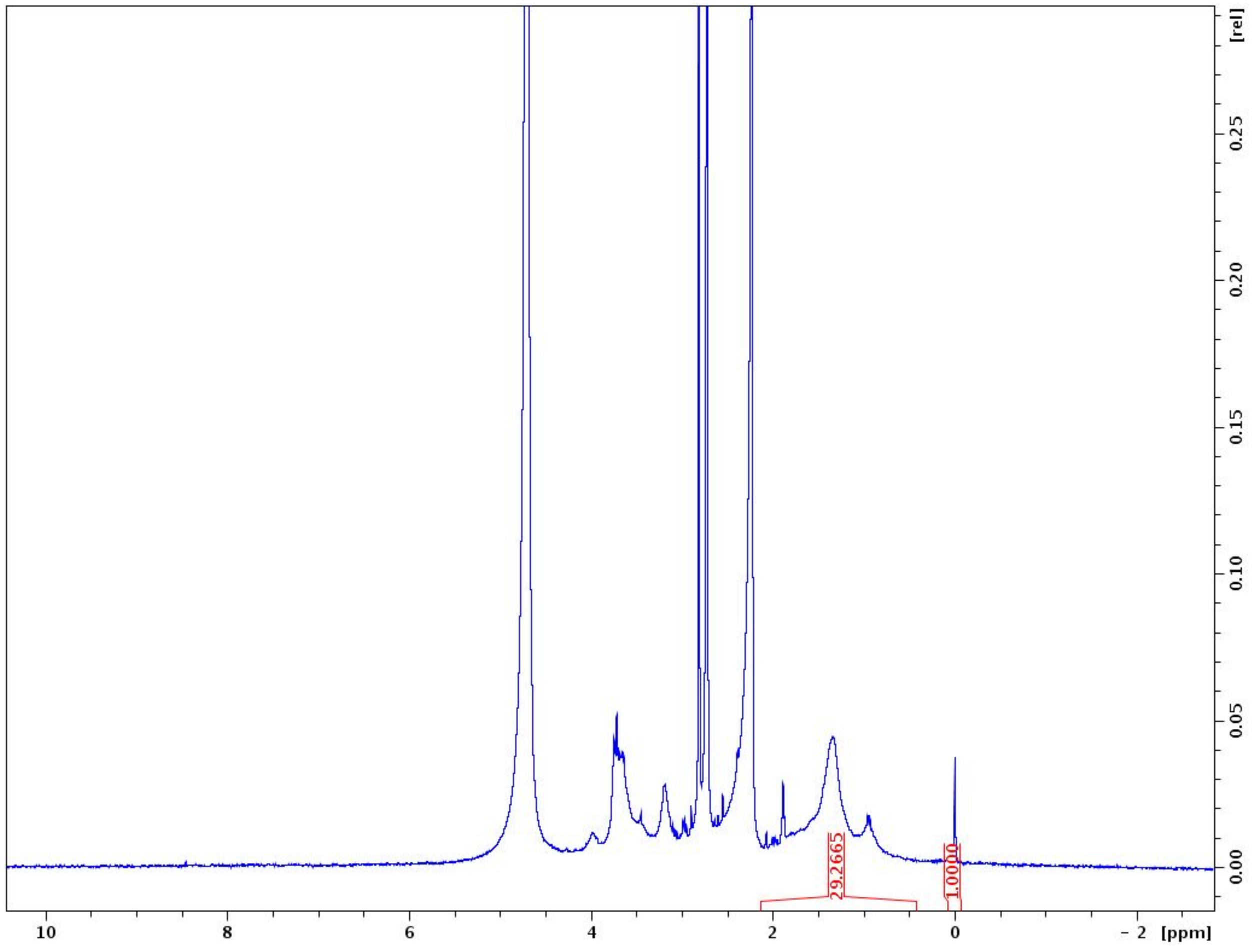

Fig. S20 ^{1}H NMR spectra of NP4 at 200mM NaCl, 304K. Deuterated trimethylsilyl propanoic acid (TMSP) (140 µM) was used as an internal reference, exhibiting a singlet at 0 ppm.

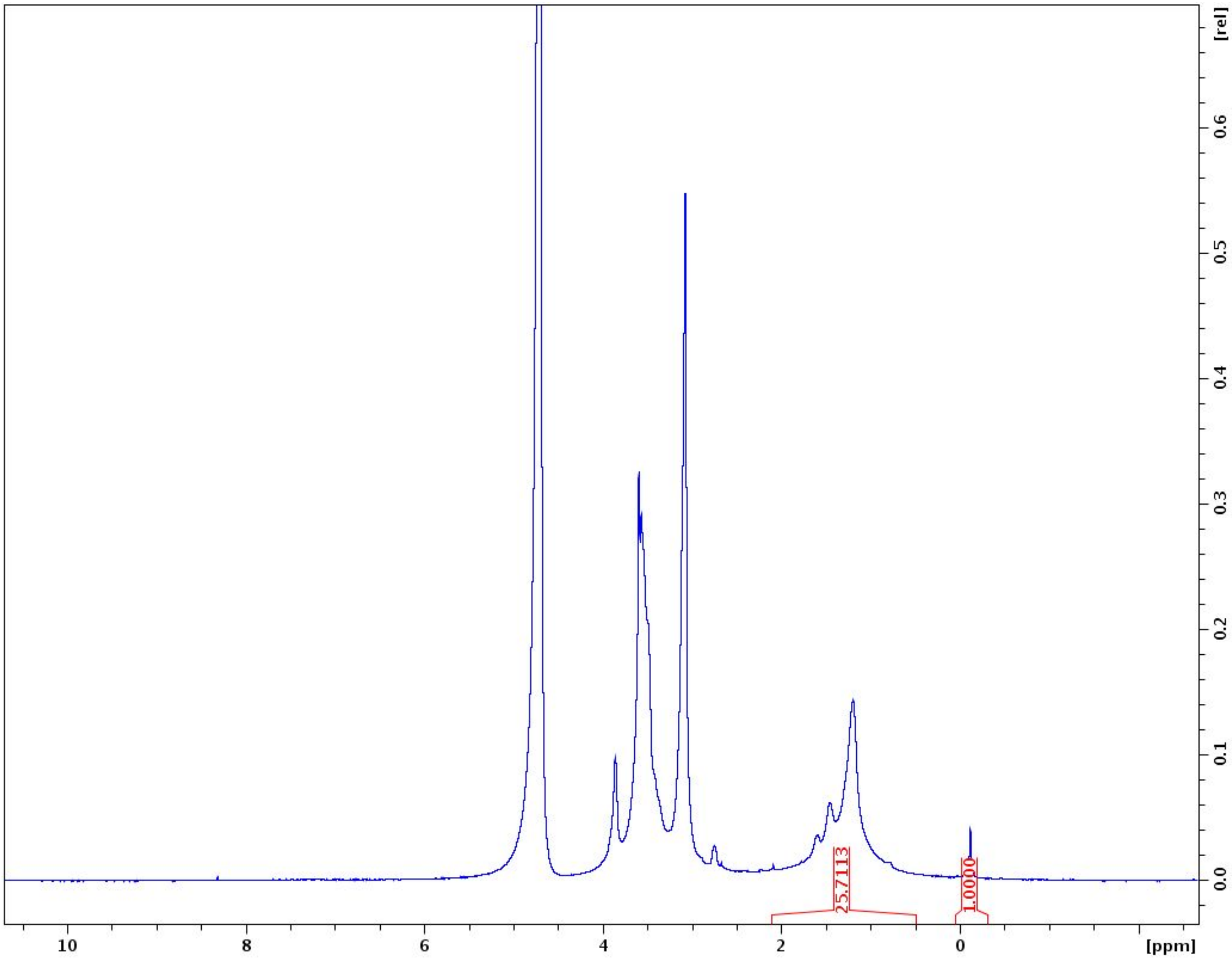

Fig. S21 ^{1}H NMR spectra of NP1 at 200mM NaCl, 310K. Deuterated trimethylsilyl propanoic acid (TMSP) (140 µM) was used as an internal reference, exhibiting a singlet at 0 ppm.

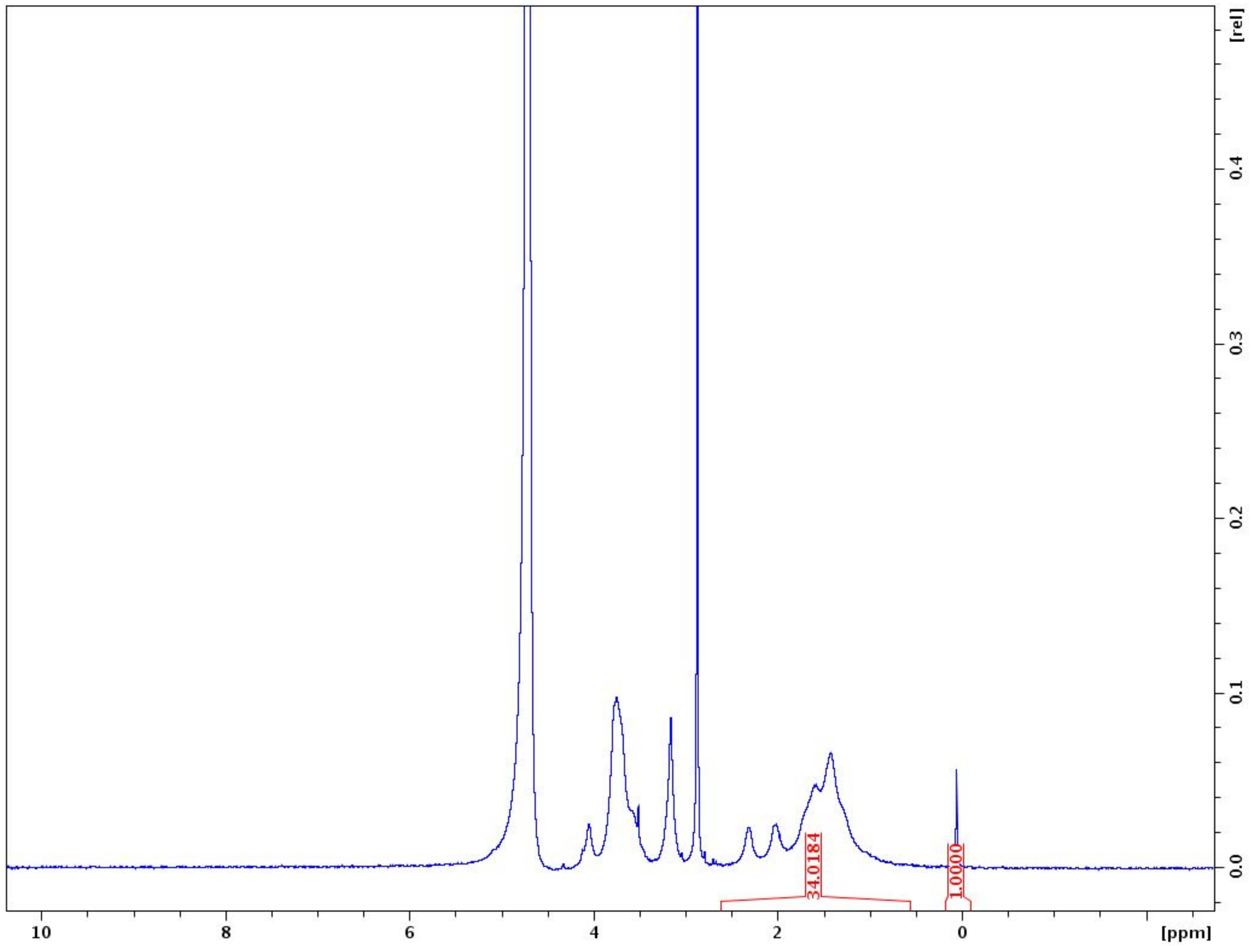

Fig. S22 ^{1}H NMR spectra of NP2 at 200mM NaCl, 310K. Deuterated trimethylsilyl propanoic acid (TMSP) (140 µM) was used as an internal reference, exhibiting a singlet at 0 ppm.

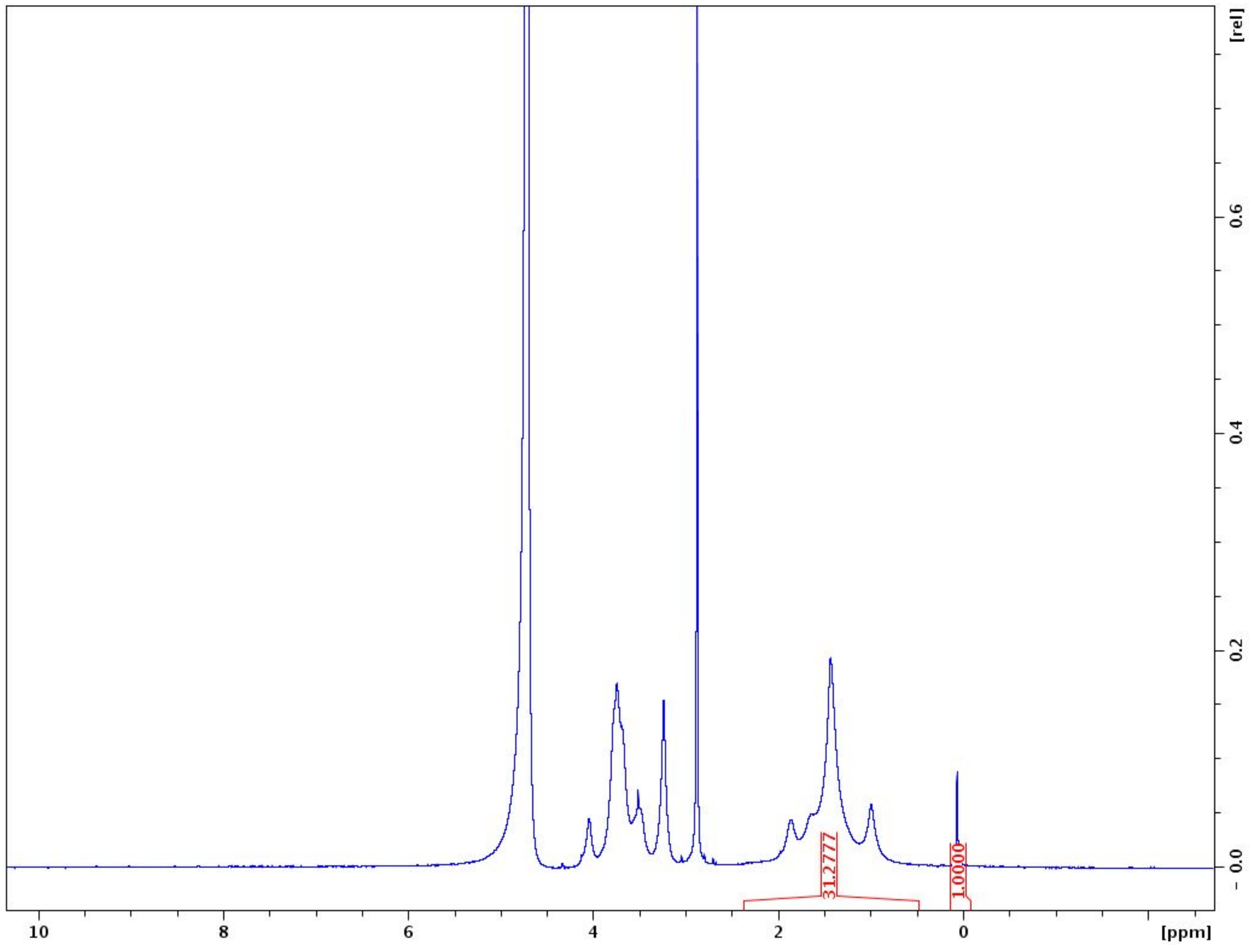

Fig. S23 ^{1}H NMR spectra of NP3 at 200mM NaCl, 310K. Deuterated trimethylsilyl propanoic acid (TMSP) (140 µM) was used as an internal reference, exhibiting a singlet at 0 ppm.

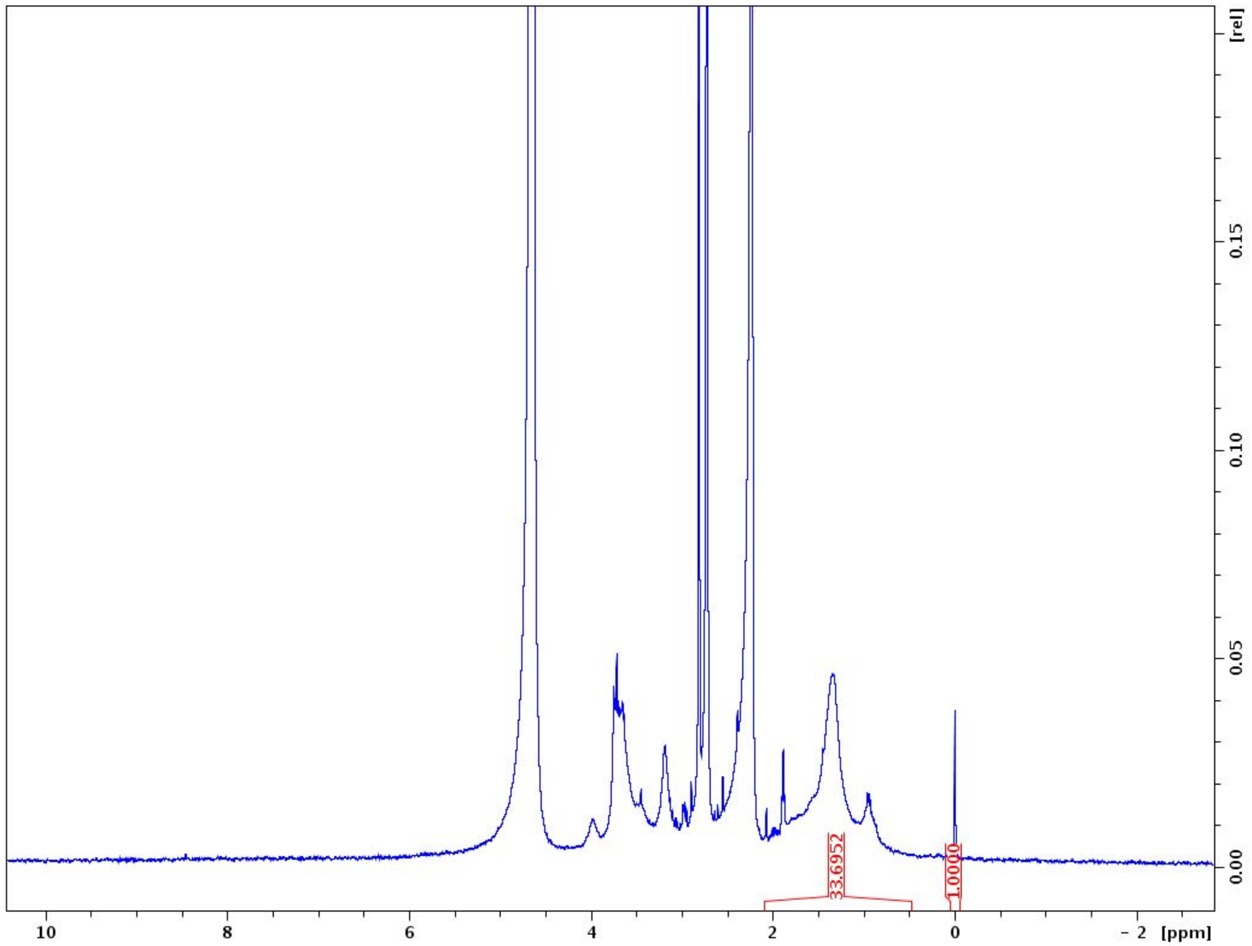

Fig. S24 ^1H NMR spectra of NP4 at 200mM NaCl, 310K. Deuterated trimethylsilyl propanoic acid (TMSP) (140 µM) was used as an internal reference, exhibiting a singlet at 0 ppm.
